# Long noncoding RNA CCDC144NL-AS1 knockdown induces naïve-like state conversion of human pluripotent stem cells

**DOI:** 10.1186/s13287-019-1323-9

**Published:** 2019-07-29

**Authors:** Yingying Wang, Baosen Guo, Zengrong Xiao, Haijun Lin, Xi Zhang, Yueqiang Song, Yalei Li, Xuehu Gao, Jinjun Yu, Zhihua Shao, Xuekun Li, Yuping Luo, Siguang Li

**Affiliations:** 1 Stem Cell Translational Research Center, Tongji Hospital, Tongji University School of Medicine, Shanghai, 200065 China; 2 College of Life Sciences, Nanchang University, Nanchang, 330031 China; 3 The Children’s Hospital, School of Medicine, Zhejiang University, Hangzhou, 310052 China; 4 Human Aging Research Institute and School of Life Science, Nanchang University, Nanchang, 330031 China; 5 Collaborative Innovation Center for Brain Science, Tongji University, Shanghai, 200092 China

**Keywords:** lncRNA, Human PSCs, Human naive pluripotency, MAPK/ERK signaling pathway, Wnt signaling pathway

## Abstract

**Background:**

Human naïve pluripotency state cells can be derived from direct isolation of inner cell mass or primed-to-naïve resetting of human embryonic stem cells (hESCs) through different combinations of transcription factors, small molecular inhibitors, and growth factors. Long noncoding RNAs (lncRNAs) have been identified to be crucial in diverse biological processes, including pluripotency regulatory circuit of mouse pluripotent stem cells (PSCs), but few are involved in human PSCs’ regulation of pluripotency and naïve pluripotency derivation. This study initially planned to discover more lncRNAs possibly playing significant roles in the regulation of human PSCs’ pluripotency, but accidently identified a lncRNA whose knockdown in human PSCs induced naïve-like pluripotency conversion.

**Methods:**

Candidate lncRNAs tightly correlated with human pluripotency were screened from 55 RNA-seq data containing human ESC, human induced pluripotent stem cell (iPSC), and somatic tissue samples. Then loss-of-function experiments in human PSCs were performed to investigate the function of these candidate lncRNAs. The naïve-like pluripotency conversion caused by CCDC144NL-AS1 knockdown (KD) was characterized by quantitative real-time PCR, immunofluorescence staining, western blotting, differentiation of hESCs in vitro and in vivo, RNA-seq, and chromatin immunoprecipitation. Finally, the signaling pathways in CCDC144NL-AS1-KD human PSCs were examined through western blotting and analysis of RNA-seq data.

**Results:**

The results indicated that knockdown of *CCDC144NL-AS1* induces naïve-like state conversion of human PSCs in the absence of additional transcription factors or small molecular inhibitors. CCDC144NL-AS1-KD human PSCs reveal naïve-like pluripotency features, such as elevated expression of naïve pluripotency-associated genes, increased developmental capacity, analogous transcriptional profiles to human naïve PSCs, and global reduction of repressive chromatin modification marks. Furthermore, CCDC144NL-AS1-KD human PSCs display inhibition of MAPK (ERK), accumulation of active β-catenin, and upregulation of some LIF/STAT3 target genes, and all of these are concordant with previously reported traits of human naïve PSCs.

**Conclusions:**

Our study unveils an unexpected role of a lncRNA, *CCDC144NL-AS1*, in the naïve-like state conversion of human PSCs, providing a new perspective to further understand the regulation process of human early pluripotency states conversion. It is suggested that CCDC144NL-AS1 can be potentially valuable for future research on deriving higher quality naïve state human PSCs and promoting their therapeutic applications.

**Electronic supplementary material:**

The online version of this article (10.1186/s13287-019-1323-9) contains supplementary material, which is available to authorized users.

## Background

Human pluripotent stem cells (PSCs), including human embryonic stem cells (ESCs) and human induced pluripotent stem cells (iPSCs), have the ability to self-renew indefinitely and possess the potential to differentiate into derivatives of all three embryonic germ layers [1–3]. Human PSCs (hPSCs) provide a unique model to study the early process of human embryogenesis, supply an investigational tool to disease modeling and drug discovery, and also could be a probable source of cells for regenerative medicine [4, 5]. Whereas, unlike mouse ESCs [6], which are derived from pre-implantation embryos and can be maintained in a naïve, ground state in vitro [7, 8], human PSCs exhibit developmentally more advanced or primed pluripotency akin to that of mouse post-implantation epiblast-derived stem cells (EpiSCs) [9–12].

Recent years’ studies demonstrate that human ESCs can be converted to the naïve state by transgene dependent or independent ways. Continuous exogenous expression of different combinations of pluripotency-associated factors, such as octamer-binding protein 4 (*OCT4*, also known as *POU5F1*) and Kruppel-like factor 4 (*KLF4*), or *KLF2* and *KLF4*, or *NANOG* and *KLF2*, allowed the derivation and expansion of human cells encompassing ground pluripotency attributes of mouse ESCs in “2i/LIF” conditions supplemented with MEK inhibitor (PD0325901), GSK3 inhibitor (CHIR99021), and human LIF [13, 14].

Short-term overexpression of *NANOG* and *KLF2* in 2i/LIF followed by t2iLGö medium containing titrated 2i with LIF and protein kinase C inhibitor (Gö6983) also allowed the derivation and maintenance of ground-state human PSCs [15]. In addition, the temporary expression of STAT3 in 2i/LIF could reprogramme human ESCs to naive-like pluripotency as well [16]. By contrast, transgene-independent human naïve pluripotency induction methods implicate in multiple small molecular inhibitors and growth factors. For instance, culture conditions containing 2i/LIF in company with inhibitors of Jun N-terminal kinase (JNK), P38, aPKC, Rho-associated protein kinase (ROCK), and growth factors FGF2 and TGFβ were described for inducing and maintaining human naïve PSCs [17]. Moreover, alternative conditions for inducing human naïve PSCs were reported, such as “3iL” condition which contained MEK inhibitor, GSK3 inhibitor, BMP inhibitor, and human LIF in mTeSR1 medium, and “5i/L/A” condition compromised of inhibitors for MEK, GSK3, ROCK, BRAF, and SRC and growth factors human LIF and activin [14, 18]. Additionally, human naïve PSCs can also be derived through directly culturing isolated cells of human inner cell mass in “t2iLGöY” medium which contained inhibitors for GSK3, MEK, PKC, and ROCK; growth factor human LIF; and ascorbic acid [19]. All artificial human naïve PSCs raise the feasibility and practical avenues to acquire an earlier pluripotency state than conventional primed human PSCs in vitro.

Previous studies of the underlying molecular mechanisms of pluripotency maintenance and lineage specification demonstrate a complex network involving transcription factors [20–24], chromatin regulators [25–28], and noncoding RNAs [29–35]. Long noncoding RNAs (lncRNAs) have been identified to be extensively transcribed in the mammalian genome [36, 37]. They are currently defined as a heterogeneous class of RNA polymerase II transcripts longer than 200 nucleotides with no apparent protein-coding potential [38]. LncRNAs show precisely regulated temporal and spatial expression patterns [39], and a large number of studies have corroborated lncRNAs’ essential roles in diverse biological processes, such as immune response, cell cycle regulation, T cell development, and nervous system development [40–43]. Likewise, lncRNAs have been validated as an integral part of mouse PSCs’ pluripotency regulatory circuit [32, 33, 35, 44]. Nevertheless, only a tiny fraction of lncRNAs were functionally characterized in the intricate network of human PSCs in contrast to hundreds of them identified in mouse PSCs [34, 45, 46]. So, we initially planned to discover more lncRNAs possibly playing significant roles in the regulation of human PSCs’ pluripotency.

Intriguingly, in our subsequent research, we identified a lncRNA, *CCDC144NL-AS1*, the downregulation of which in human PSCs induced naïve-like pluripotency conversion without exogenous factors. CCDC144NL-AS1-knockdown (KD) human PSCs exhibited a series of naïve pluripotency specific features, such as naïve-like dome-shaped clones, increased expression of naïve pluripotency-associated genes, elevated developmental capacity, analogous transcriptional profiles to human naïve PSCs, and global reduction of repressive chromatin-modifying marks. Furthermore, *CCDC144NL-AS1* knockdown in human PSCs resulted in MAPK (ERK) inhibition, β-catenin activation, and elevated expression of some LIF/STAT3 target genes, and all of these were required for naïve pluripotency maintenance. Our study unveils a lncRNA’s unexpected role in the complex conversion process of human early pluripotency states, and it can be potentially valuable for future research on deriving higher quality naïve state human PSCs.

## Methods

### Cell culture

The hESC lines H9 and H14 and hiPSC line HDF-iPS were used in this study. The cells were cultured feeder free on Matrigel (BD Biosciences) in TeSR-E8 medium (Stem Cell Technologies), and their medium was changed daily. The hPSCs were passaged with Versene (Gibco) every 5–7 days. Culture conditions of stable CCDC144NL-AS1-KD and NC hPSCs were the same as that of hPSCs. Rock inhibitor Y27632 (Merck Millipore) was suggested to be added into the culture medium for 1 day at the final concentration of 10 μM during the first few passages of CCDC144NL-AS1-KD hPSCs. Single cells of these cells were obtained through Accutase (Gibco) digestion, and Y27632 (10 μM) was added to the medium for 1 day after plating cells.

### Plasmid construction, lentivirus production, and infection

For *CCDC144NL-AS1* knockdown, shRNA sequences targeting *CCDC144NL-AS1* were cloned into the lentivirus vector GV248 (from GeneChem company) downstream of an hU6 promoter (hU6-sh-*CCDC144NL-AS1*-Ubiquitin-EGFP-IRES-puromycin). The effective target sequence of *CCDC144NL-AS1* used in this study is GTGTGGGAAGCTATAAGCATT. For the overexpression of *CCDC144NL-AS1*, *CCDC144NL-AS1* cDNA was cloned into the lentivirus vector CV084 (from GeneChem company) downstream of a Ubi promoter (Ubi-CCDC144NL-AS1-SV40-Neomycin). For the production of lentiviruses, each lentivirus vector was co-transfected with packing vectors into 293T cells, then virus supernatant was collected and concentrated through ultracentrifuge (Beckman, Avanti J-301). Single cells of hPSCs were infected with viruses in TeSR-E8 medium supplemented with 2 μg/ml polybrene (Sigma-Aldrich), and the medium was renewed 6 h after virus infection. Positively infected cells were isolated by adding 2 μg/ml puromycin (*CCDC144NL-AS1* knockdown experiments) or 400 μg/ml G418 (*CCDC144NL-AS1* overexpression experiments) into the culture medium 48 h after virus infection. The concentrations and time of antibiotics utilized should be adjusted according to different cell viability.

### RNA extraction, reverse transcription, and quantitative real-time PCR

For the expression analysis, total RNA of cells was extracted by RNAiso Plus (Takara), according to the manufacturers’ instructions. The reverse transcription was performed by PrimeScript RT Master Mix (Takara). Quantitative real-time PCR (qPCR) reactions were carried out using FastStart Universal SRBR Green Master (Roche). Primer sequences are listed in Additional file 1: Table S1. Relative expression levels of genes were calculated by ΔΔCt method normalized to *GAPDH* compared with control samples.

### Immunofluorescence staining

Cultured cells or teratoma sections were fixed with 4% paraformaldehyde in PBS for 15 min, washed with PBS for three times, and then permeabilized in 0.1% Triton X-100 (1% Triton X-100 for teratoma sections) for 30 min. After blocking in 3% donkey or goat serum for an hour, primary antibodies were incubated overnight at 4 °C. Then, corresponding secondary antibodies were incubated for an hour at room temperature. The nuclei were stained by DAPI (Roche). Antibodies are listed in Additional file 2: Table S2.

### Western blot analysis

Cells were lysed with RIPA lysis buffer supplemented with protease inhibitor cocktail (Roche) and phosphatase inhibitor cocktail (Roche). An equal amount of protein was separated on an 8% or 10% SDS-polyacrylamide gel and then was transferred to a 0.45-μm pore PVDF membrane (Merck Millipore). After being blocked in 5% skim milk for an hour, the membrane bars were incubated in primary antibodies overnight at 4 °C. Then, the membrane bars were washed with 0.1% TBST for three times and incubated in secondary antibodies for an hour at room temperature. Antibodies are listed in Additional file 2: Table S2. After being washed in 0.1% TBST three times, the protein signals were detected with an ECL Western Blotting Substrate kit (Thermo Scientific) on Amersham Imager 600 (GE Healthcare Life Sciences) system.

### G-banding karyotype analysis

Cells mostly grown under logarithmic phase were used for G-banding karyotype analysis. Confluent cells were treated with 0.2 μg/ml colchicine (Sigma-Aldrich) for 2 h and then digested into single cells with Accutase. The single cells were suspended in 0.075 mol/l potassium chloride for 25 min at 37 °C and fixed for 30 min at room temperature under a solution consisting of methyl alcohol and glacial acetic acid at the volume ratio of 2:1. Then, the karyotypes were analyzed in Da An Health Testing Center (Shanghai, China).

### Directed differentiation in vitro

Directed differentiation to neuronal cells was performed by PSC Neural Induction Medium (Life Technologies). Cells with 15–20% confluency were changed into the complete neural induction medium, including Neurobasal Medium and Neural Induction Supplement, for 7 days. On day 7, single cells of NSCs (passage 0) were harvested by utilizing Accutase and re-seeded on a Matrigel-coated plate under complete Neural Expansion Medium which included Neurobasal Medium, Advanced DMEM/F-12, and Neural Induction Supplement at the volume ratio of 49:49:2. Rock inhibitor Y27632 was added into the medium for 1 day at the final concentration of 5 μM during the first 4 passages of NSCs. Endodermal differentiation of hPSCs was performed by culturing cells under the KnockOut DMEM (Gibco) medium supplemented with 20% fetal bovine serum (FBS, Hyclone), 1% MEM NEAA (Gibco), 1% l-glutamine (Gibco), 1% penicillin-streptomycin (Gibco), 20 ng/ml Activin A (PeroTech), and 50 ng/ml BMP4 (PeproTech) for 7 days. hPSCs were differentiated into mesodermal cells with STEMdiff™ Mesoderm Induction Medium (STEMCELL). Cells were dissociated into single cells and seeded onto a Matrigel-coated 24-well plate at a density of 1 × 10^5^ cells per well under TeSR-E8 medium supplemented with rock inhibitor Y27632 (10 μM). From the next day, cells were changed into STEMdiff™ Mesoderm Induction Medium for 4 days and then harvested for subsequent analyses.

### Teratoma formation

Human PSCs grown to 70–80% confluency were scraped off the plates, centrifuged, and re-suspended in 100 μl TeSR-E8 medium per well of a 6-well plate. The 100-μl cells were used to inject subcutaneously into a dorsal flank site of a non-obese diabetic severe combined immunodeficiency (NOD/SCID) mouse. About 7–11 weeks after injection, the tumors were dissected, fixed in 4% paraformaldehyde, sectioned, and then stained by hematoxylin and eosin.

### RNA-seq library construction and sequencing

For RNA-seq, total RNA of samples was extracted by RNAiso Plus (Takara) following the product’s descriptions. RNA-seq libraries were constructed by Novogene (Beijing, China) and sequenced on Hiseq-4000 system (Illumina).

### Chromatin immunoprecipitation

Cells were incubated in 1% formaldehyde for 10 min at room temperature, and the cross-linking process was terminated by adding glycine at the final concentration of 125 mM. The cells were lysed and then digested with micrococcal nuclease (NEB) for 20 min at 37 °C. After the cells were sonicated, the extracts were immunoprecipitated by antibodies against H3K4me3 (ab8580, Abcam) or H3K27me3 (39155, ACTIVE MOTIF) overnight at 4 °C. The extracts were then incubated in magnetic beads for 2 h at 4 °C, and the beads were washed. Finally, the DNA products were eluted and purified. ChIP-seq libraries were constructed by Novogene (Beijing, China) and sequenced with the Novaseq-PE150 system (Illumina).

### Public datasets

The datasets (GSE60945, E-MTAB-2031, E-MTAB-4461) used in this study, including three human reset PSC samples, three 3iL hESC samples, six conventional primed hPSC samples, and nine HNES samples, were downloaded from GEO database (http://www.ncbi.nlm.nih.gov/geo/) and ArrayExpress database (http://www.ebi.ac.uk/arrayexpress).

### Read mapping and transcripts assembly of RNA-seq data

Reads of RNA-seq were aligned to human reference genome (hg19) by the spliced read aligner Hisat2 (vision 2.1.0) [47]. The gene abundances were estimated and normalized to fragments per kilobase of transcript per million fragments mapped (FPKM) using StringTie (version 2.2.1) [48] with default settings, and read counts of genes were summarized by HTSeq (vision 0.7.2) [49].

### Weighted gene co-expression network analysis

Weighted gene co-expression network analysis (WGCNA) [50] in R package was used in this study to construct the weighted gene co-expression network of multiple samples. A major step in the module-centric calculation is to cluster genes of similar interconnection expression patterns into network modules. To construct a signed weighted co-expression network, we first calculate the adjacency matrix, which preserves the underlying continuous correlation instead of a dichotomizing one, of weighted Pearson correlation of all pair-wise genes with an index *β* = 20. Then, we use the topological overlap measure (TOM) as proximity, which is used as an input of hierarchical clustering, to combine the correlation between pair-wise genes with that of these two genes share with other “third party” genes, and this algorithm conforms to the actual situation of intergenic relationships in biological pathways.

### Statistical analysis, gene function enrichment analysis, and gene set enrichment analysis

Differential expression analysis of genes in this study was identified by R Bioconductor package DESeq2 [51], and genes with adjusted *p* value < 0.05 were considered as differentially expressed. ClusterProfiler [52] in R was used for GO and KEGG term gene function enrichment analysis and visualization. The significance test of prior defined functional gene set between genes expressed by different groups of samples was performed by gene set enrichment analysis (GSEA) [53, 54].

### ChIP-seq data analysis

The ultrafast, memory-efficient read aligner bowtie2 (vision 2.2.6) [55] was used to align the reads of ChIP-seq to the human reference genome (hg19). The output of bowtie2 was sorted and index by SAMtools [56]. Peak calling was performed by MACS2 (version 2.1.1.20160309). DeepTools [57] was used for the alignment of reads as input and normalized to reads per kilobase and million mapped reads (RPKM) and computing signals over a set of regions from 3 kb upstream of the TSS to 3 kb downstream of the TES of all genes and developmental genes. The signal visualization of interested genes was conducted by DeepTools and Integrative Genome Viewer (IGV) [58]. Read count frequency of regions which range from 3 kb upstream to 3 kb downstream of the TSS was performed by ChIPseeker [59].

## Results

### Candidate lncRNAs tightly correlated with human pluripotency were screened through bioinformatic and experimental approaches

**Fig. 1:**
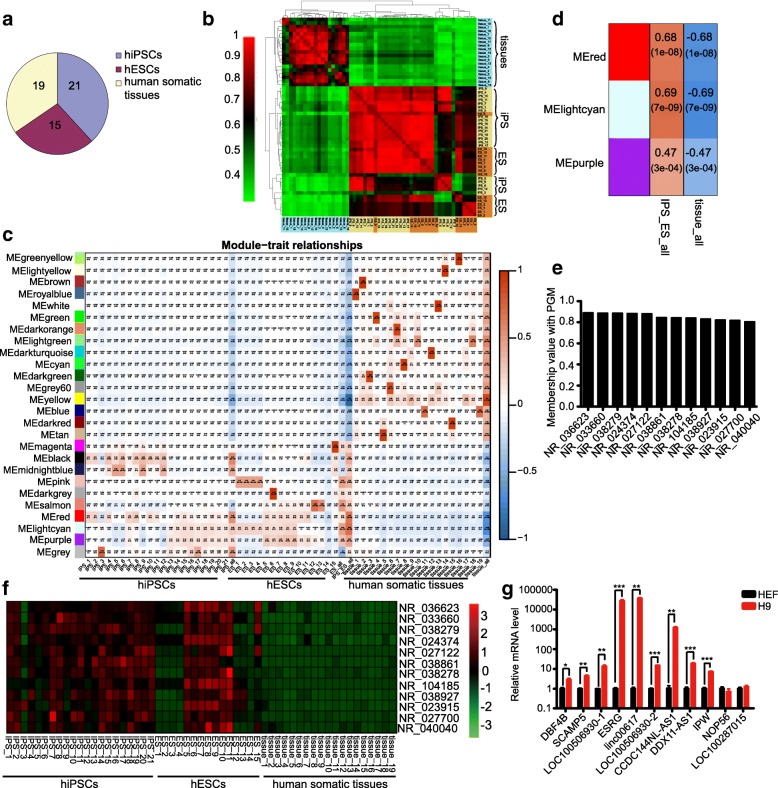
Screening of lncRNAs that are tightly correlated with human pluripotency. **a** Constituent numbers of RNA-seq data used in this study to screen candidate lncRNAs. **b** Unsupervised hierarchical clustering of 55 RNA-seq data from hiPSC (yellow), hESC (orange), and human somatic tissue (cyan) samples (FPKM ≥ 0.1). **c** Sample-specific modules identified by WGCNA. Two figures of each square respectively represent the correlation between a module and corresponding sample, and *p* value of the correlation value. Colors of squares correspond to different correlation types: positive correlation (red), negative correlation (blue), no correlation (white). **d** Top three human PSC-specific modules identified by WGCNA. **e** Twelve noncoding genes showing membership values above 0.8 with PGM module. **f** Expression of filtered 12 noncoding genes, which were identified by RNA-seq data of 55 samples. **g** Quantitative RT-PCR analyses of 11 lncRNAs’ expression levels in H9 and HEF cells. Error bars indicate SEM (*n* = 3). *p < .05; **p < .01; ***p < .001

We initially attempted to characterize lncRNAs that support pluripotency maintenance of human PSCs. To screen lncRNAs that were specifically expressed in human PSCs, we collected a total of 55 RNA-seq data, which contained 21 human iPSC (hiPSC) samples, 15 human ESC (hESC) samples, and 19 human somatic tissue samples, from GEO datasets (Fig. 1a, Additional file 3: Table S3). Unsupervised hierarchical clustering of the 55 RNA-seq data (FPKM ≥ 0.1) demonstrated that despite occasional intersections, all 21 hiPSC and 15 hESC samples clustered together and were clearly separated from the 19 human somatic tissues (Fig. 1b).

Next, all 55 RNA-seq data were subjected to weighted gene co-expression network analyses (WGCNA). We obtained multiple gene network modules representing properties of different human somatic tissues and identified top 3 notable modules (MEred, MElightcyan, and MEpurple) shared by all human PSC samples (Fig. 1c, d; Additional file 4: Figure S1a). Gene Ontology (GO) and Kyoto Encyclopedia of Genes and Genomes (KEGG) analyses of these three modules revealed that their functions were primarily associated with RNA metabolism and processing, DNA replication and repair, chromatin organization and modification, oocyte meiosis and cell cycle, and protein catabolic processes (Additional file 4: Figure S1b-d).

To obtain candidate lncRNAs tightly correlated with human pluripotency, we took 32 pluripotency-associated genes as a module (PGM) (Additional file 4: Figure S1e) and calculated the membership values for all noncoding genes from MEred, MElightcyan, and MEpurple modules with it. Then, 12 lncRNAs whose membership values were above 0.8 stood out (Fig. 1e). The transcriptional levels of these 12 lncRNAs in most human PSC samples were obviously higher than that in somatic tissues according to RNA-seq data (Fig. 1f). Furthermore, we performed qPCR experiments to examine their expression in one human ESC line (H9) and human embryonic fibroblast (HEF) cells. Leaving out a pseudogene NR_024374, nine lncRNAs were validated to be significantly highly expressed in H9 cells relative to HEF cells (Fig. 1g). It should be noted that functions of five validated lncRNAs were already annotated, such as *DBF4B* and *LINC00617*(*TUNAR*), the former played an important role in DNA replication and cell proliferation, and the latter was tested to be essential in maintaining pluripotency and the neural differentiation of embryonic stem cells [35, 60, 61]. The encompassment of known lncRNAs for pluripotency maintenance in our result suggested that our method was effective. Therefore, we focused on the remaining four uncharacterized lncRNAs in the following experiments, including *LOC100506930*-1 (NR_038278), *LOC100506930*-2 (NR_038279), *CCDC144NL-AS1* (NR_104185), and *DDX11-AS1* (NR_038927).

### *CCDC144NL-AS1* knockdown in human PSCs drives them to a naïve-like pluripotency state

**Fig. 2:**
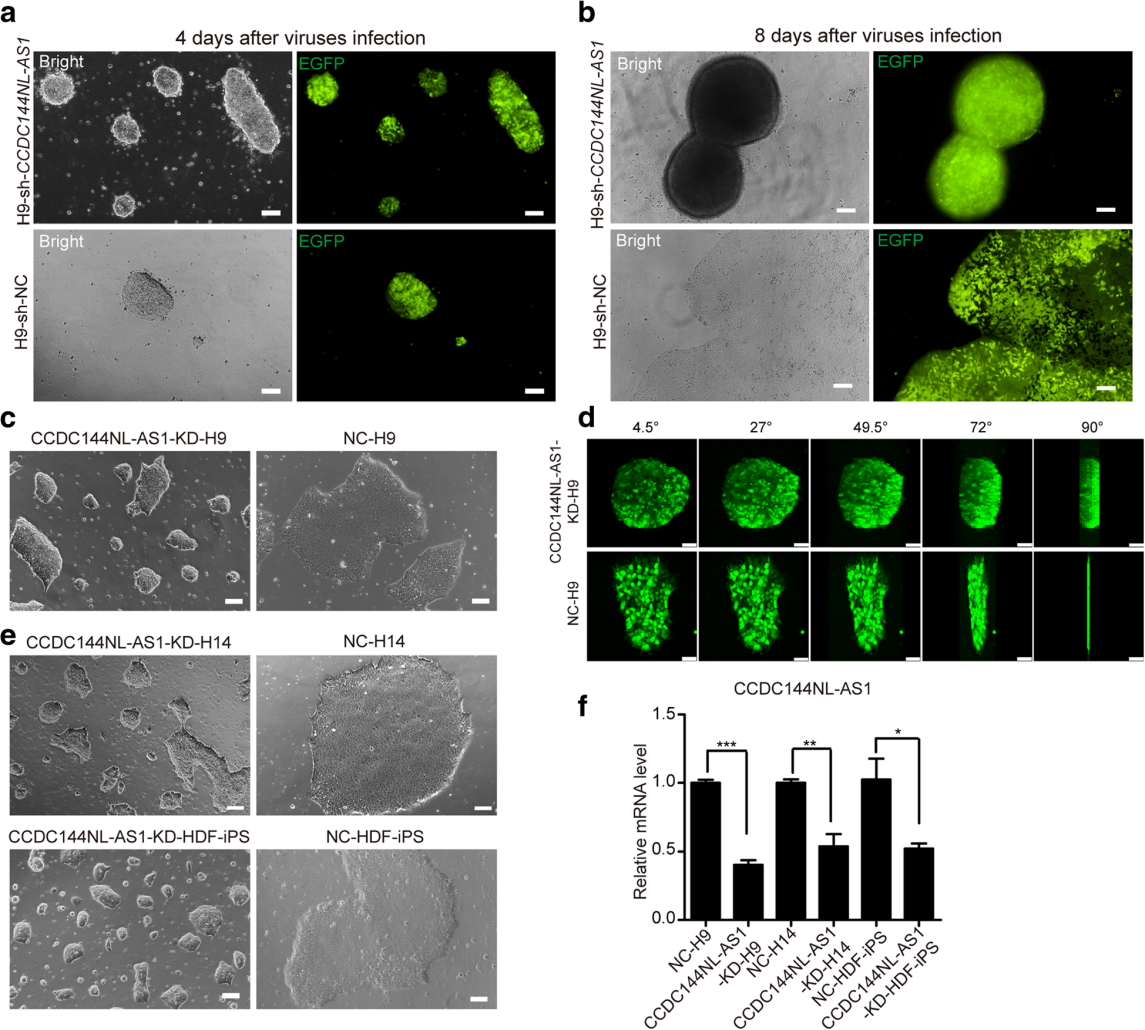
*CCDC144NL-AS1* knockdown endows human PSCs with naïve-like dome-shaped clusters. **a** Bright-field (left) and fluorescence (right) images of H9 cells infected with sh-*CCDC144NL-AS1* or sh-NC viruses on day 4. ShRNA expression plasmids used in this study contain an EGFP expression element, so EGFP fluorescence intensity reflects the virus infection efficiency of cells. Scale bars, 100 μm. **b** Bright-field (left) and fluorescence (right) images of H9 cells infected with sh-*CCDC144NL-AS1* or sh-NC viruses on day 8. Scale bars, 100 μm. **c** Representative images of stable CCDC144NL-AS1-KD-H9 and NC-H9 cells. Scale bars, 100 μm. **d** Five representative confocal EGFP fluorescence images capturing the rotation process of CCDC144NL-AS1-KD-H9 or NC-H9 colonies. Scale bars, 50 μm. The values of rotation angles are indicated above the images. **e** Representative images of CCDC144NL-AS1-KD-H14, NC-H14 cells, CCDC144NL-AS1-KD-HDF-iPS, and NC-HDF-iPS cells. Scale bars, 100 μm. **f** Quantitative RT-PCR analyses of *CCDC144NL-AS1*’s expression in CCDC144NL-AS1-KD hPSCs and their control cells. Error bars indicate SEM (*n* = 3). *p < .05; **p < .01; ***p < .001

To functionally identify the potential roles of four lncRNAs in regulating human PSCs’ pluripotency, we designed short hairpin RNAs (shRNAs) for them. At first, we focused on CCDC144NL-AS1, whose expression in H9 cells was hundreds-fold higher than that in HEF (Fig. 1g). When we infected human ES cell line H9 cells with shRNA-expressing viruses, we found that the expression of shRNA targeting *CCDC144NL-AS1* drove the edges of H9 flat clones to curl up on day 4 (Fig. 2a). And the clones were further rolled up, even completely became spheres on day 8 (Fig. 2b). It should be noted that the cells needed to be passaged between day 4 and day 8 to avoid excessive cell death. We passaged the cells and obtained stable CCDC144NL-AS1-KD-H9 and NC-H9 cells after several rounds of puromycin resistance screening (Fig. 2c). The effectively decreased RNA levels of *CCDC144NL-AS1* in CCDC144NL-AS1-KD-H9 cells were confirmed by qPCR analyses (Fig. 2f). From the above morphology of ES cells, we can see that the downregulation of *CCDC144NL-AS1* drove H9 to naïve-like dome-shaped colonies from the primed flat disc ones. We also performed confocal layer scanning of these two different cluster types accompanied by 90° horizontal rotation (Additional file 5: Video S1 and Additional file 6: Video S2.). The videos and five representative images from each group clearly exhibited that CCDC144NL-AS1-KD-H9 clusters were much more compact and thicker than single flat layers of NC-H9 (Fig. 2d). For H9 is a female human ES cell line, we therefore examined whether *CCDC144NL-AS1* knockdown would engender similar effects in a male human ES cell line (H14) and a male human iPS cell line (HDF-iPS) derived from human dermal fibroblasts. Likewise, we observed primed-to-naïve-like morphology changes in H14 and HDF-iPS cells after *CCDC144NL-AS1* knockdown (Fig. 2e, f).

**Fig. 3:**
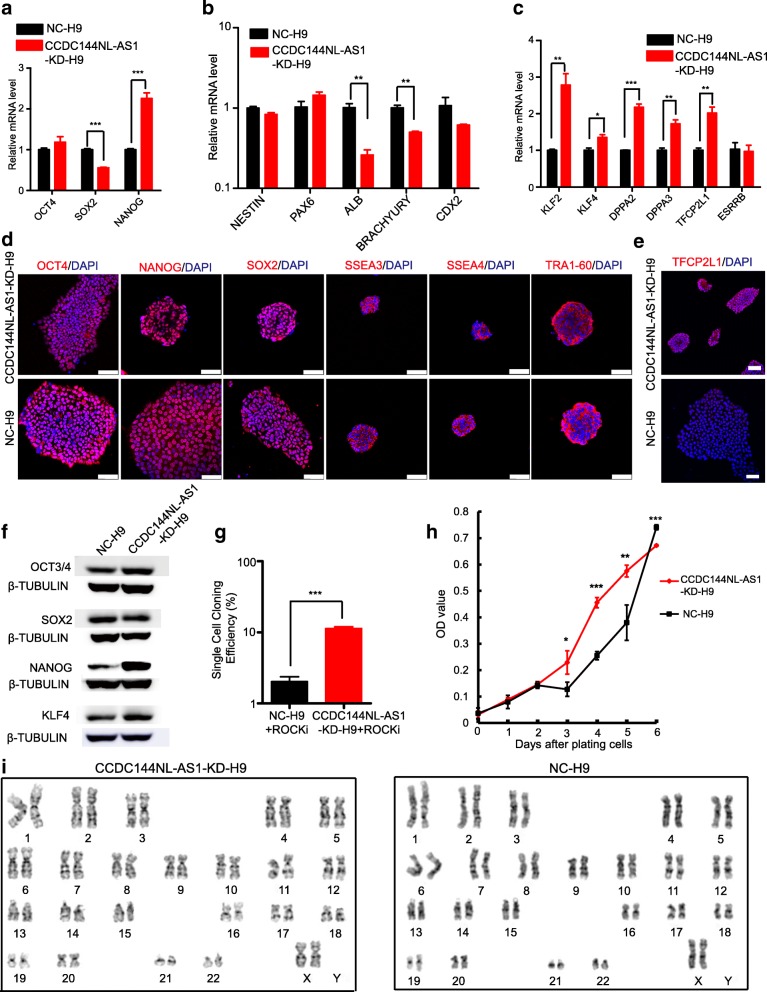
Naïve-like pluripotency validation of *CCDC144NL-AS1*-downregulated human ESCs. Quantitative RT-PCR analyses of three core pluripotency genes (**a**), lineage commitment factor genes (**b**), and naïve pluripotency-associated genes (*c*) in CCDC144NL-AS1-KD-H9 and NC-H9 cells. Error bars indicate SEM (*n* = 3). *p < .05; **p < .01; ***p < .001. Immunostaining images of pluripotency-associated markers OCT4, NANOG, SOX2, SSEA3/4, and TRA-1-60 (**d**) and naïve pluripotency-specific marker TFCP2L1 (**e**). Scale bars, 75 μm. **f** Western blot detection of naïve pluripotency-related transcription factors, such as NANOG and KLF4, and shared pluripotency transcription factors, such as OCT4 and SOX2, in CCDC144NL-AS1-KD-H9 and NC-H9 cells. β-Tubulin was used as an endogenous control. **g** Single-cell cloning efficiencies of CCDC144NL-AS1-KD-H9 and NC-H9 cell lines. Error bars indicate SEM (n = 3); ***, *p* < .001. **h** Growth curves of CCDC144NL-AS1-KD-H9 and NC-H9 cell lines. Error bars indicate SEM (*n* = 3). *p < .05; **p < .01; ***p < .001. **i** Karyotype analysis of CCDC144NL-AS1-KD-H9 and NC-H9 cell lines

Next, we performed a series of experiments to characterize the pluripotent state of CCDC144NL-AS1-KD-H9 cells. Our qPCR analyses revealed that the expression of core pluripotency factors *OCT4*, *SOX2*, and *NANOG* changed differently in CCDC144NL-AS1-KD-H9 cells. *SOX2* was significantly decreased, and *NANOG* was markedly elevated and no obvious changes in *OCT4* levels (Fig. 3a). Among them, *NANOG* was considered essential for the establishment of naïve pluripotency [62]. We also examined the expression levels of lineage commitment factors and found that both endodermal marker *ALB* and mesodermal marker *BRACHYURY* were evidently decreased in CCDC144NL-AS1-KD-H9 cells relative to NC-H9 cells (Fig. 3b). Furthermore, we observed obviously elevated expression of naïve pluripotency-associated transcription factors, such as *KLF2*, *KLF4*, *DPPA2*, *DPPA3*, and *TFCP2L1* (Fig. 3c). In addition, CCDC144NL-AS1-KD-H9 cells stained positive for pluripotency markers OCT4, SOX2, NANOG, SSEA3, SSEA4, TRA-1-60, and naïve pluripotency-specific TFCP2L1 (Fig. 3d, e). And western blot results further corroborated the expression changes of SOX2, NANOG, and KLF4 at the protein level (Fig. 3f). In addition, we also examined the expression levels of these genes in CCDC144NL-AS1-KD-H14 and CCDC144NL-AS1-KD-HDF-iPS cells. In CCDC144NL-AS1-KD-H14 cells, we observed obviously increased expression of naïve pluripotency-associated genes *NANOG*, *KLF2*, *DPPA2*, *DPPA3*, and *TFCP2L1* and evidently decreased neuroectodermal marker *PAX6* and trophectoderm marker *CDX2* (Additional file 7: Figure S2a, b). In CCDC144NL-AS1-KD-HDF-iPS cells, we observed obviously increased *NANOG*, *KLF2*, *KLF4*, *DPPA2*, *DPPA3*, and *TFCP2L1* and decreased neuroectodermal marker *PAX6* and endodermal marker *ALB* (Additional file 7: Figure S2c, d). Furthermore, both of these cells stained positive for pluripotency markers OCT4, SOX2, NANOG, SSEA3, SSEA4, TRA-1-60, and naïve pluripotency-specific TFCP2L1 (Additional file 7: Figure S2e, f). And western blot results further validated the expression changes of NANOG and KLF4 in these cells (Additional file 7: Figure S2 g).

We then measured the single-cell cloning efficiency of these cells and found that CCDC144NL-AS1-KD-H9, CCDC144NL-AS1-KD-H14, and CCDC144NL-AS1-KD-HDF-iPS cells presented much higher single-cell cloning efficiency than control cells (Fig. 3g, Additional file 8: Figure S3 a). Although the absolute values are not identical to those in previous reported human naïve PSCs, possibly owing to the differences in cell culture and technical systems in the analyses, the upregulated trend relative to the primed PSCs is similar. Naïve PSCs are characterized by high rates of proliferation, so we performed cell growth curve analysis. From our growth curve results, we can see that CCDC144NL-AS1-KD-H9 cells showed obviously increased OD values, which reflected elevated cell numbers, from day 3 to day 5 than NC-H9 cells, but they were surmounted by control cells on day 6 (Fig. 3h). Remarkably, CCDC144NL-AS1-KD-H9 cells entered the exponential phase of growth earlier than NC-H9 cells; however, we suppose, their gradually increasing layers of cells piled up within limited space led to lowered cell growth on day 6. Whereas, primed H9 cells maintained a single layer growth pattern from the beginning to end in this experiment. The growth curves indicate that *CCDC144NL-AS1* downregulation drives H9 cells to proliferate faster, and the tendency is concordant with the naïve pluripotency property. Additionally, we assessed the cell cycle on day 4 after plating the cells. CCDC144NL-AS1-KD-H9 cells revealed obviously increased S+G2/M phase cells relative to the control, indicating that they grow faster than primed cells (Additional file 8: Figure S3 b-c). Whereas, CCDC144NL-AS1-KD-H14 and CCDC144NL-AS1-KD-HDF-iPS did not reveal remarkably increased S+G2/M phase cells than control, suggesting that they did not grow much faster than control cells. Besides, both CCDC144NL-AS1-KD-H9 and NC-H9 cells maintained a normal karyotype (Fig. 3i).

It should be clear that the naïve degree of published human naïve ESCs generated from different conditions varies from each other. Obviously, CCDC144NL-AS1-KD-H9 cells own the naïve-like colonies, express higher naïve pluripotency-related genes, and possess naïve-like cell growth patterns, despite that the naïve pluripotency degree of them is somewhat restricted.

### CCDC144NL-AS1-downregulated human PSCs exhibit elevated developmental potential in vitro and in vivo

**Fig. 4:**
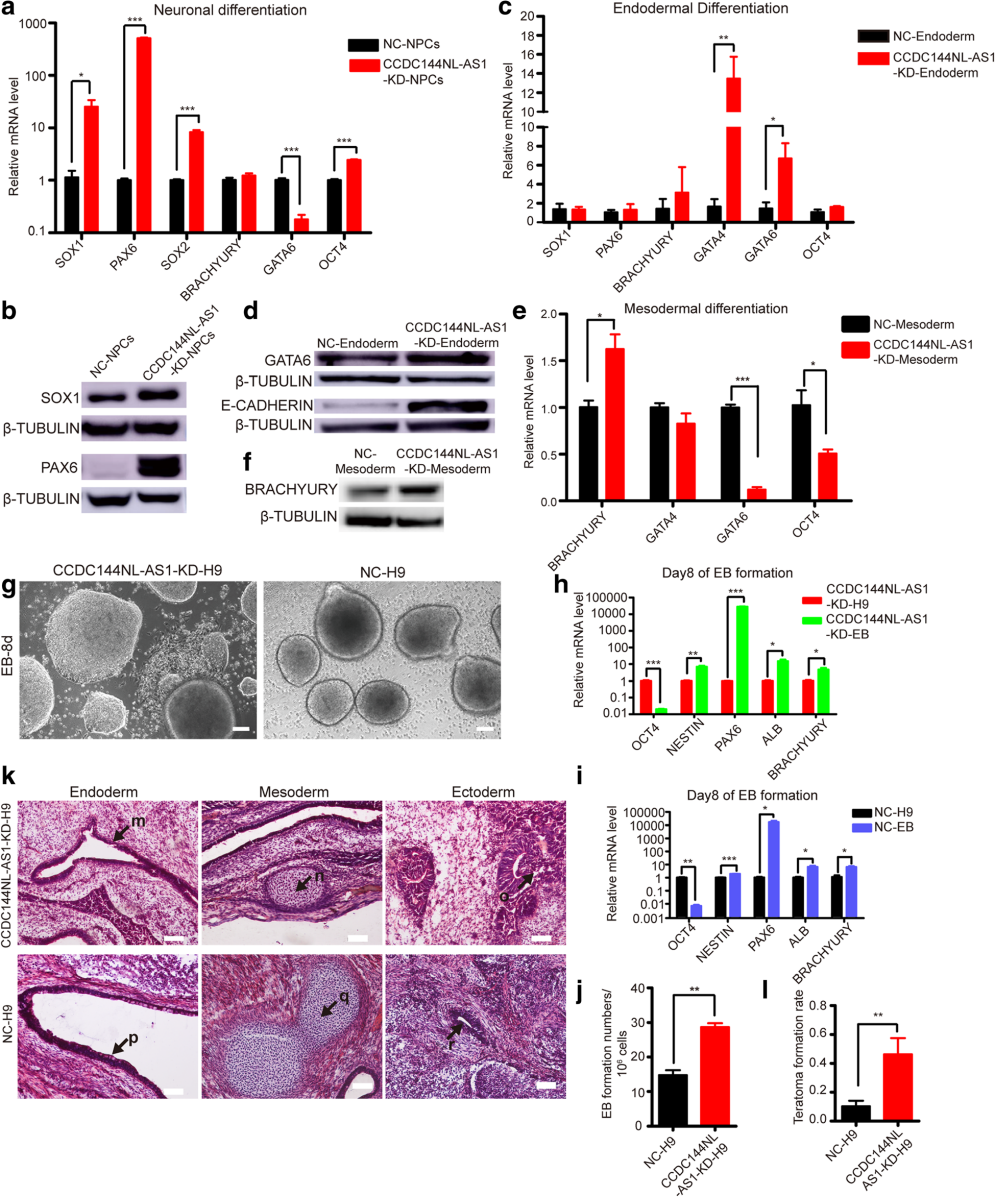
Developmental potential of CCDC144NL-AS1-downregulated human ESCs in vitro and in vivo. Quantitative RT-PCR analyses of expression levels of lineage-specific genes in CCDC144NL-AS1-KD-H9- and NC-H9-derived neuronal (**a**), endodermal (**c**), and mesodermal (**e**) cells. Error bars indicate SEM (*n* = 3). *p < .05; **p < .01; ***p < .001. Western blot analyses of lineage-specific genes’ expression in CCDC144NL-AS1-KD-H9- and NC-H9-derived neuronal (**b**), endodermal (**d**), and mesodermal (**f**) cells. β-Tubulin was used as an endogenous control. **g** Morphologies of embryoid bodies differentiated from CCDC144NL-AS1-KD-H9 and NC-H9 cells. Scale bars, 100 μm. Quantitative RT-PCR analyses of lineage-related genes in embryoid bodies generated from CCDC144NL-AS1-KD-H9 (**h**) and NC-H9 (**i**) cells. Error bars indicate SEM (*n* = 3). *p < .05; **p < .01; ***p < .001. **j** Embryoid body numbers generated from per 10^6^ CCDC144NL-AS1-KD-H9 and NC-H9 cells. Error bars indicate SEM (*n* = 3). **p < .01. **k** Hematoxylin and eosin staining of teratomas containing tissues of all three embryonic germ layers derived from CCDC144NL-AS1-KD-H9 and NC-H9 cells. (m, p) Gut-like epithelium (endoderm). (n, q) Cartilage (mesoderm). (o, r) Neural tissue (ectoderm). Scale bars, 500 μm. **l** Teratoma formation rate of CCDC144NL-AS1-KD-H9 and NC-H9 cells. Error bars indicate SEM (*n* = 13). **p < .01

Some human naïve induction medium endowed their naïve PSCs with more efficient differentiation abilities [18, 63]. To investigate the differentiation capacity of our CCDC144NL-AS1-KD-H9 cells in vitro, we directed them and control cells towards neuronal, endodermal, and mesodermal cells under lineage-specific differentiation medium (Additional file 9: Figure S4a). Quantitative PCR analyses revealed that CCDC144NL-AS1-KD-H9-derived neural precursor cells (NPCs) expressed much higher levels of neuronal/neuroectodermal-specific marker genes *SOX1*, *PAX6*, and *SOX2* and much lower levels of endodermal-specific gene *GATA6* compared with control cells (Fig. 4a), illustrating that *CCDC144NL-AS1* downregulation endued H9 cells with more homogeneity and higher efficiency towards neuronal differentiation. Western blot analyses further confirmed the elevated SOX1 and PAX6 protein levels in CCDC144NL-AS1-KD-H9-derived NPCs (Fig. 4b). Upon differentiation of endoderm, we also observed efficient differentiation potential of *CCDC144NL-AS1* knockdown H9 cells relative to their control counterparts, with RNA levels of endodermal-specific *GATA4* and *GATA6* significantly increased (Fig. 4c) and protein levels of endodermal-specific E-CADHERIN and GATA6 remarkably elevated (Fig. 4d). On mesodermal differentiation, CCDC144NL-AS1-KD-H9-derived mesodermal cells expressed obviously higher levels of mesodermal-specific gene *BRACHYURY* and lower levels of endodermal-specific gene *GATA6* relative to control cells (Fig. 4e). It was hard to detect the expression of neuroectodermal-specific *SOX1* and *PAX6*, because both groups expressed very low levels of them. In addition, mesodermal-specific BRACHYURY was verified higher in protein levels in CCDC144NL-AS1-KD-H9-derived mesodermal cells compared with control cells (Fig. 4f). Both results demonstrated that *CCDC144NL-AS1*-downregulated H9 cells differentiated to mesodermal cells with more homogeneity and higher efficiency.

Furthermore, we also assessed the differentiation capacity of CCDC144NL-AS1-KD-H14 and CCDC144NL-AS1-KD-HDF-iPS cells in vitro. Upon neuroectoderm differentiation, CCDC144NL-AS1-KD-HDF-iPS showed much more efficient differentiation capacity with obvious higher neuronal/neuroectodermal-specific genes *SOX1*, *PAX6*, and *SOX2* expression relative to their control cells (Additional file 9: Figure S4 b,c), while the expression levels of *SOX1*, *PAX6*, and *SOX2* in CCDC144NL-AS1-KD-H14-derived NPCs were lower than those in their counterparts (Additional file 9: Figure S4 d,e). On endodermal differentiation, in accordance with CCDC144NL-AS1-KD-H9, both of these two cells revealed evidently increased endodermal-specific *GATA4* and *GATA6* gene expression compared with control cells (Additional file 9: Figure S4 f-i). When differentiated into mesodermal cells, CCDC144NL-AS1-KD-H14 showed significantly elevated expression of mesodermal-specific *BRACHYURY* relative to the control (Additional file 9: Figure S4 j-k), while CCDC144NL-AS1-KD-HDF-iPS cells did not reveal increased mesodermal differentiation compared with control cells (Additional file 9: Figure S4 l-m). The results above indicated that when directed differentiation in vitro, human PSCs may response differently due to *CCDC144NL-AS1* downregulation.

Next, we performed in vitro spontaneous differentiation of CCDC144NL-AS1-KD-H9 and NC-H9 cells by generating embryoid bodies (EBs). After 8 days of suspending ES clones in TeSR-E6 differentiation medium, we obtained EBs that expressed significantly upregulated marker genes of three germ layers (Fig. 4g–i). It is noteworthy that there were always some EBs being attached to the plate bottom at day 8 in CCDC144NL-AS1-KD-H9 cells (Fig. 4g, left), whereas control cells were not (Fig. 4g, right). We speculated that the much more compact cell mass of CCDC144NL-AS1-KD-H9 endowed them with stronger propensities to form clones. In addition, we also examined the expression levels of some important cell adhesion molecules and found that CCDC144NL-AS1-KD-H9-derived EBs expressed evidently higher cell adhesion-associated genes *E-CAD*, *ZO-1*, *CTNNA1*, *MUC1*, and *DSG2* than NC-H9-derived EBs, in accordance with the phenomenon we observed (Additional file 10: Figure S5 a). Moreover, CCDC144NL-AS1-KD-H9 cells displayed markedly elevated levels in our assay of EB formation numbers per 10^6^ cells in contrast with control cells (Fig. 4j). This result was concordant with those of high rates of proliferation and increased single-cell cloning efficiencies of CCDC144NL-AS1-KD-H9 cells (Fig. 3g, h), all of which reflected their higher cell viability. We also evaluated the EB formation ability of CCDC144NL-AS1-KD-H14, CCDC144NL-AS1-KD-HDF-iPS, and their counterparts. All of them could form EBs, and CCDC144NL-AS1-KD-H14 and CCDC144NL-AS1-KD-HDF-iPS showed significantly increased EB numbers per 10^6^ cells relative to control cells (Additional file 10: Figure S5 b-g).

For differentiation in vivo, human CCDC144NL-AS1-KD-H9 and NC-H9 cells were injected subcutaneously into NOD/SCID mice for teratoma formation. Both groups obtained teratomas containing tissues from all three germ layers (Fig. 4k, Additional file 11: Figure S6 a, b), whereas H9 cells whose *CCDC144NL-AS1* expression was downregulated showed significantly elevated teratoma formation rate, suggesting that the downregulation of *CCDC144NL-AS1* improved in vivo developmental capacity of H9 cells (Fig. 4l). For CCDC144NL-AS1-KD-H14, CCDC144NL-AS1-KD-HDF-iPS, and their control cells, we also performed teratoma formation experiments. All of them could obtain teratomas containing tissues from all three germ layers (Additional file 11: Figure S6 c). In accord with the CCDC144NL-AS1-KD-H9 cells, CCDC144NL-AS1-KD-H14 and CCDC144NL-AS1-KD-HDF-iPS revealed obviously elevated teratoma formation rate (Additional file 11: Figure S6 d,e).

In summary, CCDC144NL-AS1-KD-H9 cells exhibited higher efficiency in directed differentiation to neuroectoderm, endoderm, and mesoderm relative to control cells. Although CCDC144NL-AS1-KD-H14 and CCDC144NL-AS1-KD-HDF-iPS cells behaved differently in some directed differentiation aspect, lowered expression of *CCDC144NL-AS1* rendered human pluripotent stem cells to differentiate more efficiently in spontaneous EB formation in vitro and teratoma formation in vivo.

### The transcriptome of CCDC144NL-AS1-knockdown human PSCs distinguishes from that of conventional human PSCs and resembles those of human naïve PSCs

**Fig. 5:**
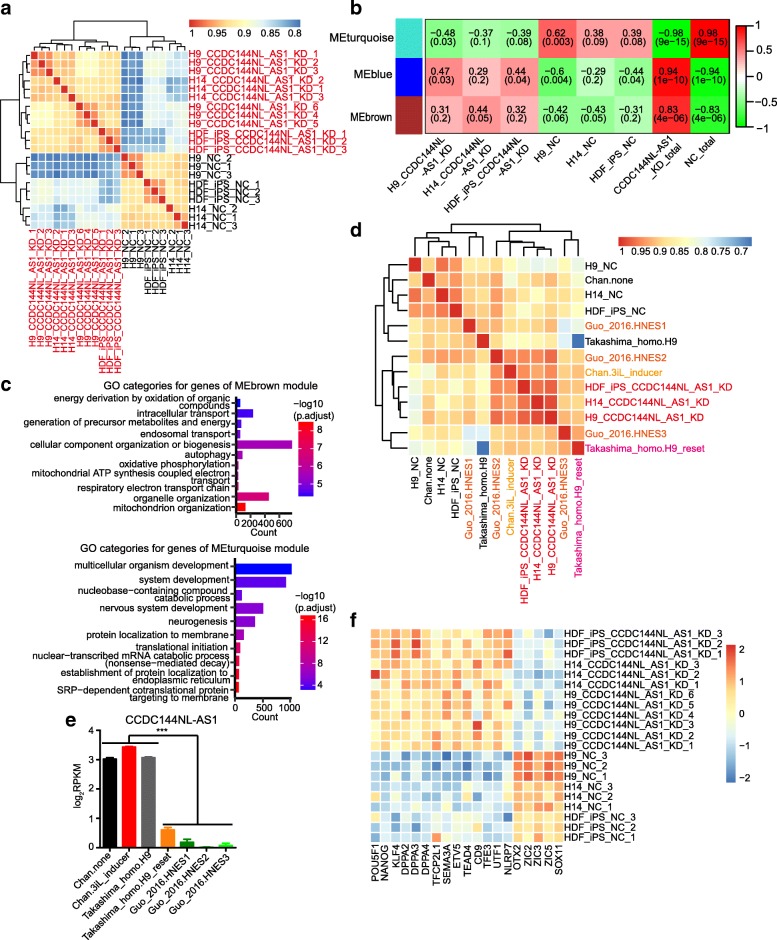
The transcriptome characteristics of CCDC144NL-AS1-knockdown hPSCs. **a** Unsupervised hierarchical clustering of RNA-seq data from CCDC144NL-AS1-KD-H9, CCDC144NL-AS1-KD-H14, CCDC144NL-AS1-KD-HDF-iPS, and their corresponding negative control cells. CCDC144NL-AS1-KD hPSC sample names are indicated in red, and all control sample names are indicated in black. **b** Three sample-specific modules identified by WGCNA. Two figures of each square respectively represent the correlation between a module and a corresponding sample, and *p* value of the correlation value. Colors of squares correspond to different correlation types: positive correlation (red), negative correlation (green), no correlation (white). **c** Gene Ontology (GO) analyses of MEbrown (upper panel) and MEturquoise (lower panel) module genes. **d** Unsupervised hierarchical clustering of RNA-seq data from CCDC144NL-AS1-KD hPSC, negative control hPSC, and samples of three other studies Chan et al. [18], Takashima et al. [15], and Guo et al. [19]. Sample names from different experiments are indicated in different colors, and all control sample names are indicated in black. **e** The expression data of *CCDC144NL-AS1* in naïve hPSCs from Chan et al. [18], Takashima et al. [15], and Guo et al. [19] and in control hPSCs. Displayed are log_2_ RPKM values. **f** Expression of genes identified by others as associating with the human naïve and primed states in CCDC144NL-AS1-KD hPSCs and control cells. The expression levels are determined by RNA-seq data.

To gain more insights into the molecular features of *CCDC144NL-AS1*-downregulated human PSCs, we assessed the transcriptional profiles of CCDC144NL-AS1-KD-H9 cells, CCDC144NL-AS1-KD-H14 cells, CCDC144NL-AS1-KD-HDF-iPS cells, and their corresponding negative control cells using RNA-seq. Unsupervised hierarchical clustering of these samples revealed that they were clearly separated into two groups due to *CCDC144NL-AS1* knockdown despite of different cell lines (Fig. 5a).

We next conducted WGCNA to detect gene co-expression network modules of CCDC144NL-AS1-KD human PSCs. Obviously, two gene co-expression modules, MEbrown and MEblue, showed the highest correlation with total CCDC144NL-AS1-KD hPSC samples (Fig. 5b). On the other side, MEturquoise presented high correlation with negative control hPSC samples and low correlation with CCDC144NL-AS1-KD human PSCs (Fig. 5b). GO classification and enrichment analyses exhibited that MEbrown module involved terms mainly about mitochondrion organization, respiratory electron transport chain, oxidative phosphorylation, autophagy, cellular component organization or biogenesis, and intracellular transport (Fig. 5c, upper panel). MEblue was associated with RNA processing, RNA splicing, and cellular macromolecule metabolic process (Additional file 12: Figure S7a). It is noteworthy that the terms, like “oxidative phosphorylation” and “respiratory electron transport chain,” enriched in CCDC144NL-AS1-KD cells were consistent with previous observations in mouse ESCs and human naïve PSCs, whereas mouse EpiSCs and human ESCs relied principally on aerobic glycolysis in energy generation [64–66]. Our gene set enrichment analysis (GSEA) further validated the upregulated enrichment of CCDC144NL-AS1-KD-H9 cells for respiratory chain (Additional file 12: Figure S7b). On the other hand, MEturquoise module, which was negatively correlated with CCDC144NL-AS1-KD cells, unveiled GO categories related to SRP-dependent cotranslational protein targeting to the membrane, nuclear-transcribed mRNA catabolic process, translational initiation, neurogenesis, nervous system development, and system development (Fig. 5c, lower panel).

Afterwards, we compared the transcriptional profiles of our CCDC144NL-AS1-KD human PSCs with those of previously reported human naïve PSCs. We selected three groups of sequencing data coming from Chan et al. [18], Takashima et al. [15], and Guo et al. [19]. Unsupervised hierarchical clustering of these samples and ours revealed that CCDC144NL-AS1-KD hPSCs preferred to cluster with the naïve datasets of Chan et al. and Takashima et al. and HNES2 and HNES3 samples from Guo et al., while conventional PSCs showed high correlation with the primed parts (Fig. 5d). Naïve cells from Takashima et al. were demonstrated as one of the representative naïve cell types similar to human pre-implantation embryos [67]. Human naïve embryonic stem (HNES) cells from Guo et al. were generated directly from isolated cells of human inner cell mass and cultured in “t2iLGöY” medium. However, we can also see that HNES1 from Guo et al. was intermixed with conventional PSCs, and the other two HNES cell lines were closer to the naïve parts (Fig. 5d). This may have resulted from the internal individual properties of HNES1 samples. Additionally, we examined the expression pattern of *CCDC144NL-AS1* in these previously reported human naïve PSCs and found that *CCDC144NL-AS1* was obviously downregulated in naïve cells from Takashima et al. and Guo et al. but not in naïve cells from Chan et al. (Fig. 5e). This result suggested that the relatively lower expression level of *CCDC144NL-AS1* might be an important aspect for human naïve PSCs which were more similar to human pre-implantation embryos.

Next, in agreement with the expression patterns of genes associated with human naïve pluripotency, our RNA-seq data confirmed significantly elevated levels of naïve hallmarks in CCDC144NL-AS1-KD hPSCs relative to control cells, such as *NANOG*, *KLF4*, *DPPA2*, *DPPA3*, *DPPA4*, *TFCP2L1*, and *SEMA3A* (Fig. 5f). Meanwhile, the obviously reduced expression of primed pluripotency regulators such as Zinc finger of the cerebellum (*ZIC*) protein family transcription factors, *ZIC2*, *ZIC3*, *ZIC5*, and other lineage-priming factors, *OTX2* and *SOX11*, were also observed in CCDC144NL-AS1-KD hPSCs compared with control cells [68, 69] (Fig. 5f).

Generally speaking, *CCDC144NL-AS1* downregulation endowed human PSCs with distinct transcriptome characteristics from the conventional ones, and more importantly, CCDC144NL-AS1-KD human PSCs exhibited a lot of resemblances with human naïve PSCs.

### CCDC144NL-AS1-downregulated human ESCs possess some epigenetic characteristics similar to naïve pluripotency

**Fig. 6:**
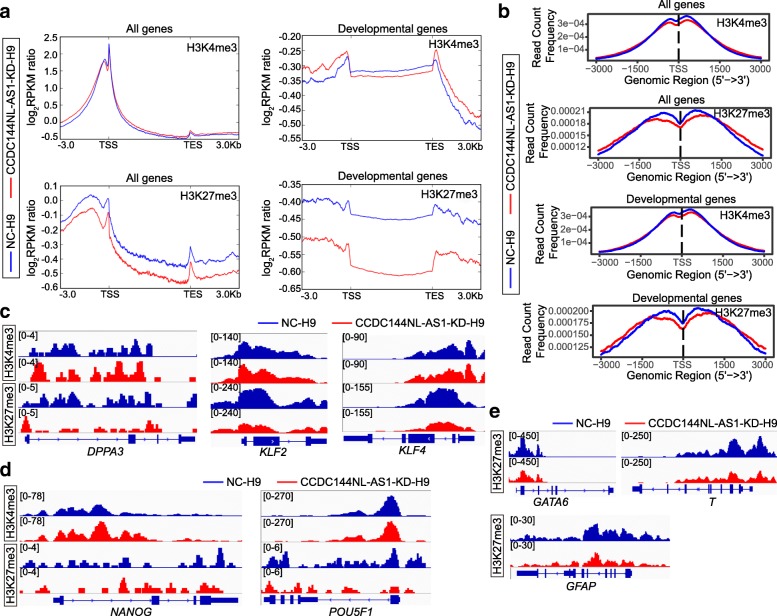
The epigenetic landscape of CCDC144NL-AS1-downregulated human ESCs. **a** Average H3K4me3 and H3K27me3 signals at all RefSeq and developmental genes in CCDC144NL-AS1-KD-H9 and NC-H9 cells. Log_2_RPKM ratio: log_2_(RPKM_sample_/RPKM_input_). **b** Read count frequency of H3K4me3 and H3K27me3 signals across all RefSeq and developmental genes in CCDC144NL-AS1-KD-H9 and NC-H9 cells. ChIP-Seq tracks for H3K4me3 and H3K27me3 in CCDC144NL-AS1-KD-H9 and NC-H9 cells at genes associated with naïve pluripotency (**c**) and core pluripotency (**d**). **e** ChIP-Seq tracks for H3K4me3 and H3K27me3 in CCDC144NL-AS1-KD-H9 and NC-H9 cells at genes related to the development

To evaluate the epigenetic profiles of CCDC144NL-AS1-KD-H9 cells, we performed chromatin immunoprecipitation followed by deep sequencing (ChIP-Seq) analyses of trimethylation of histone 3 lysine 4 (H3K4me3) and trimethylation of histone3 lysine 27 (H3K27me3), which marked the transcriptional active and silencing of chromatin state, respectively. Compared to negative control H9 cells, the genome-wide signals of H3K4me3 showed no obvious change in CCDC144NL-AS1-KD-H9 cells, whereas a significant reduction of genome-wide H3K27me3 signals was observed in CCDC144NL-AS1-KD-H9 cells (Fig. 6a). In addition, the decrease of H3K27me3 signals was also found over developmental genes in CCDC144NL-AS1-KD-H9 cells as compared to that in control cells (Fig. 6a). This epigenetic pattern of these two chromatin marks in CCDC144NL-AS1-KD-H9 cells showed similarities to that in human naïve stem cells [14]. The read count frequency of H3K4me3 signals across the global and developmental genes both revealed that there were slight rises over transcription start site (TSS) nearby the regions, but no change over the gene body or intergenic regions in CCDC144NL-AS1-KD-H9 cells compared with control cells (Fig. 6b). The reductions were more obvious for H3K27me3 read count frequency over TSS nearby regions of global and developmental genes in CCDC144NL-AS1-H9 cells relative to control cells (Fig. 6b).

We then examined the histone methylation profile at different gene loci. Naïve pluripotency-associated genes, such as *DPPA3*, *KLF2*, and *KLF4*, revealed apparently decreased distribution of H3K27me3 in CCDC144NL-AS1-KD-H9 cells as compared to that in control cells (Fig. 6c). The downregulation of H3K27me3 signals in CCDC144NL-AS1-KD-H9 cells was also observed at the loci of core pluripotency factors *OCT4* (*POU5F1*) and *NANOG* (Fig. 6d). On the contrary, H3K4me3 exhibited no obvious change at most of these gene loci, except for enhanced signals at *DPPA3* and *NANOG* (Fig. 6c, d). Developmental genes in human primed PSCs were known as bivalent genes marked by the emergence of H3K4me3 and H3K27me3 simultaneously. In CCDC144NL-AS1-KD-H9 cells, genes involved in the development, such as *GATA6*, *T*, and *GFAP*, revealed evidently decreased H3K27me3 deposition relative to NC-H9 cells, just like they do in human naïve PSCs relative to the primed (Fig. 6e).

The above results illustrated that although the epigenetic profiles of CCDC144NL-AS1-KD-H9 cells are not the same as human naïve PSCs, such as not remarkably higher H3K4me3 signals at most naïve pluripotency-associated genes, downregulation of *CCDC144NL-AS1* indeed triggered a genome-wide decrease of suppressive chromatin modifications in H9 cells, which was similar to human naïve PSCs.

### Rescued expression of CCDC144NL-AS1 partially sets back the naïve-like state transition of human ESCs triggered by CCDC144NL-AS1 downregulation

**Fig. 7:**
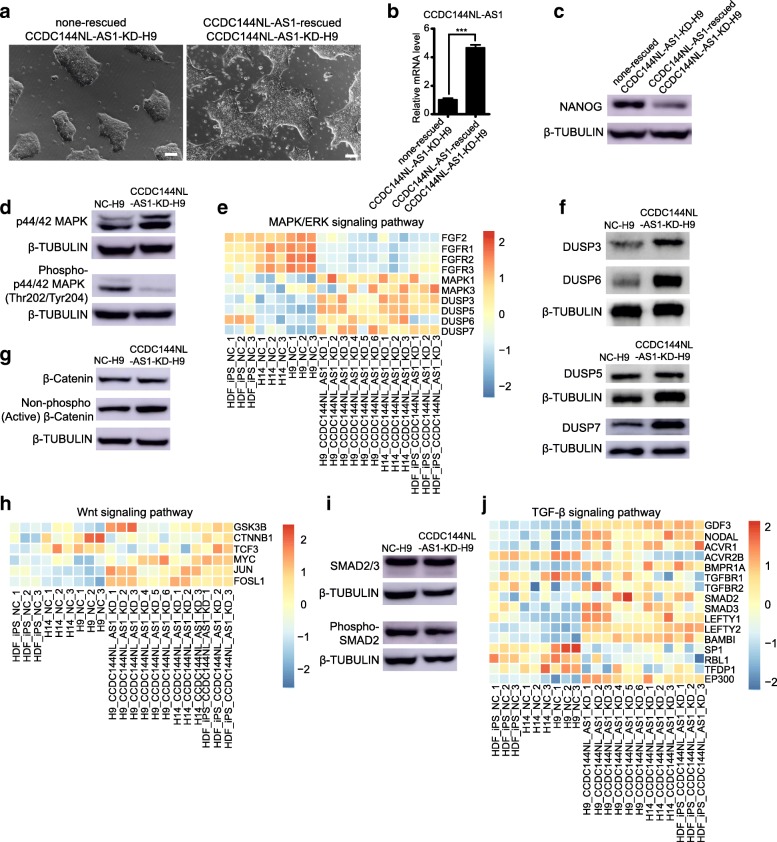
Rescued knockdown of *CCDC144NL-AS1* in CCDC144NL-AS1-KD hESCs and the signaling pathway state of CCDC144NL-AS1-KD hPSCs. **a** Morphologies of CCDC144NL-AS1-KD-H9 clusters before and after expression of *CCDC144NL-AS1* was rescued. Scale bars, 100 μm. **b** qPCR validation of *CCDC144NL-AS1*’s rescued expression in CCDC144NL-AS1-KD-H9 cells. CCDC144NL-AS1-KD-H9 cells infected with empty overexpression viruses were used as control cells. Error bars indicate SEM (*n* = 3). ***p < .001. **c** Western blot analyses of naïve pluripotency-related NANOG protein levels in CCDC144NL-AS1-rescued CCDC144NL-AS1-KD-H9 cells and CCDC144NL-AS1-KD-H9 cells infected with empty overexpression viruses were used as control cells. **d** Western blot analyses of total and phosphorylated protein levels of MAPK in CCDC144NL-AS1-KD-H9 and NC-H9 cells. **e** Heatmap of genes involved in MAPK/ERK signaling pathway in CCDC144NL-AS1-downregulated hPSCs and control cells. **f** Western blot analyses of DUSP3, DUSP6, DUSP5, and DUSP7 protein levels in CCDC144NL-AS1-KD-H9 and NC-H9 cells. **g** Western blot analyses of total and unphosphorylated (active) β-catenin protein levels in CCDC144NL-AS1-KD-H9 and NC-H9 cells. **h** Heatmap of genes involved in the Wnt signaling pathway in CCDC144NL-AS1-downregulated hPSCs and control cells. **i** Western blot analyses of total SMAD2/3 and phosphorylated SMAD2 protein levels in CCDC144NL-AS1-KD-H9 and NC-H9 cells. **j** Heatmap of genes involved in the TGF-β signaling pathway in CCDC144NL-AS1-downregulated hPSCs and control cells. The expression levels of genes in heatmaps are determined by RNA-seq data. β-Tubulin was used as an endogenous control in all western blot analyses

Next, we sought to investigate whether rescued expression of *CCDC144NL-AS1* in CCDC144NL-AS1-KD hESCs could set back the naïve-like conversion of the primed. When we rescued the expression of *CCDC144NL-AS1* in CCDC144NL-AS1-KD-H9 cells by infecting the cells with *CCDC144NL-AS1*-overexpression viruses, we observed the partial recovery of primed flat-disc colony morphology from naïve-specific dome-like clusters (Fig. 7a). CCDC144NL-AS1-KD-H9 cells infected with empty viruses were used as control cells. The rescued expression of *CCDC144NL-AS1* was confirmed by qPCR analysis (Fig. 7b). What is more, obviously reduced NANOG protein level was observed along with the primed pluripotency recovery (Fig. 7c, Additional file 13: Figure S8a). Our results indicated that the naïve-like conversion of primed hESCs indeed resulted from downregulation of *CCDC144NL-AS1*, and the conversion was partially reversible.

### CCDC144NL-AS1-downregulated human PSCs demonstrate signaling pathway dependency analogous to naïve pluripotency

Previous studies reveal that naïve pluripotency state possesses specific signaling pathway patterns distinct from the primed state [70], so we investigated some naïve-specific signaling pathways in *CCDC144NL-AS1*-downregulated human PSCs. First of all, we investigated two pathways, including MAPK/ERK signaling pathway and canonical Wnt signaling pathway. These two pathways were involved in almost all previously published protocols to induce or maintain human naïve pluripotency by utilizing two small molecular kinase inhibitors (2i) [13–15, 17, 18, 65]. One inhibitor is PD0325901 (PD), which inhibits the activity of mitogen-activated protein kinase kinase (MAPKK, MEK) and therefore gives rise to the suppression of MAPK/ERK signaling cascade; the other is CHIR99021 (CHIR), which targets glycogen synthase kinase-3 (GSK3) and results in augmented activity of Wnt signaling.

Although we observed obviously increased protein levels of total MAPK (ERK) in CCDC144NL-AS1-KD-H9 cells compared with NC-H9 cells, the active phospho-MAPK levels were greatly reduced (Fig. 7d), suggesting that the MAPK/ERK signaling was inhibited in CCDC144NL-AS1-KD-H9 cells. In line with this, the expression levels of MAPK/ERK signaling pathway-related genes fibroblast growth factor 2 (*FGF2*) and fibroblast growth factor receptors (*FGFR1*, *FGFR2*, and *FGFR3*) were much lower in CCDC144NL-AS1-KD hPSCs than those in control cells (Fig. 7e). As to the reasons why phosphorylation levels of MAPK were reduced despite the elevated total MAPK levels, we speculated that the phosphorylation process was inhibited through some particular way. In effect, we discovered the upregulation of a group of dual-specificity phosphatase (DUSP) family members from RNA-seq data (Fig. 7e). The protein levels of DUSP3, DUSP6, and DUSP7 were validated to be obviously higher in CCDC144NL-AS1-KD-H9 cells than those in control (Fig. 7f). DUSPs can dephosphorylate the threonine and the tyrosine residue sites of MAPKs and lead to their inactivation [71].

CCDC144NL-AS1-KD-H9 cells also showed increased protein levels of active unphosphorylated β-catenin relative to NC-H9 cells, indicating the higher activation degree of the Wnt signaling pathway (Fig. 7g). In consistent with the above, the expression of transcription factor 3 (*TCF3*) was reduced in CCDC144NL-AS1-KD-H9 and CCDC144NL-AS1-KD-H14 cells compared with control cells according to the RNA-seq data (Fig. 7h). TCF3 is a transcriptional repressor of pluripotency factors and β-catenin can directly abrogate TCF3 repression on the pluripotency network, so the inhibition of TCF3 is a necessary downstream effect of Wnt signaling [72, 73]. In addition, the expression levels of Wnt target genes, such as *MYC*, *JUN*, and *FOSL1*, were elevated in CCDC144NL-AS1-KD hPSCs relative to the control, further validating the stimulated Wnt signaling pathway (Fig. 7h).

Our CCDC144NL-AS1-KD hPSCs were derived and maintained with TeSR-E8 medium. TeSR-E8 is supplemented with basic fibroblast growth factor (bFGF) and TGF-β, these two components were confirmed to play indispensable roles in sustaining primed pluripotency in mouse EpiSCs and human ESCs, but were considered unnecessary for mouse ground state pluripotency maintenance [70]. Whereas in some human naïve PSCs induction methods, bFGF and TGF-β were added to their culture medium [14, 17, 65, 74], possibly implying the requirements for growth factors to stabilize human naïve PSCs. We then explored the TGF-β signaling pathway state in our cells. Western blot analysis revealed that phospho-SMAD2 protein levels were slightly decreased in CCDC144NL-AS1-KD-H9 cells compared with NC-H9 cells (Fig. 7i). Nevertheless, when we added a selective activin receptor inhibitor SB 431542, which could effectively block TGF-β signaling without toxicity [75], to the culture medium at the final concentration of 10 μM to examine the TGF-β signaling pathway dependency of our cells, we found both CCDC144NL-AS1-KD-H9 cells and NC-H9 cells underwent differentiation in 7 days (Additional file 13: Figure S8b). These results indicated that CCDC144NL-AS1-KD-H9 cells could not tolerate the TGF-β signaling pathway blockage caused by SB 431542. RNA-seq data demonstrated that TGF-β signaling pathway ligands *GDF3* and *NODAL*; receptors *ACVR1*, *BMPR1A*, and *TGFBR2*; and signal transducers *SMAD2* and *SMAD3* expressed higher levels in CCDC144NL-AS1-KD hPSCs relative to control cells (Fig. 7j). At the same time, there were two receptors *ACVR2B* and *TGFBR1* being downregulated and three negative regulators *LEFTY1*, *LEFTY2*, and *BAMBI* being elevated in these cells, reflecting the simultaneous existence of a stimulation state and a suppressive feedback loop of TGF-β signaling pathway in CCDC144NL-AS1-KD hPSCs than control cells (Fig. 7j). Consistent with the intricate state, TGF-β signaling target genes *SP1*, *RBL1*, and *TFDP1* expressed lower levels and another *EP300* expressed higher levels in CCDC144NL-AS1-KD hPSCs compared with control cells (Fig. 7j).

Another nonnegligible signaling pathway implicated in the naïve pluripotency sustaining is the LIF/STAT3 signaling pathway. Reinforcement of STAT3 activity in combination with 2i/LIF can revert human PSCs from the primed pluripotency to a naïve-like pluripotency state [16]. We also analyzed the expression levels of STAT3 target genes and found that eight target genes (*ICAM1*, *IER3*, *ZFP36L1*, *SULF1*, *SPRY2*, *SMAD7*, *PIM3*, *DACT1*) were upregulated in CCDC144NL-AS1-KD hESCs compared to control cells, implying the relatively high activity of STAT3 signaling (Additional file 13: Figure S8c), whereas a few STAT3 target genes were not significantly reduced in CCDC144NL-AS1-KD-HDF-iPSCs, and this might be caused by individual properties of this iPS cell line (Additional file 13: Figure S8c).

It was reported that naïve human ESCs induced by small chemical molecules relied on mTORC2 subunit to maintain pluripotency [76]. So, we also analyzed the mTORC2 dependency of CCDC144NL-AS1-KD-H9 cells. We added rapamycin (mTORC1 inhibitor) and PP242 (mTORC1/2 inhibitor) to the culture medium of the cells separately. Both of CCDC144NL-AS1-KD-H9 and NC-H9 cells exhibited apoptosis in rapamycin and PP242 condition (Additional file 13: Figure S8d). After 48 h treatment, the cells were fixed and immunofluorescence stained with OCT4 and NANOG. The results showed that the expression of NANOG in CCDC144NL-AS1-KD-H9 was significantly reduced after rapamycin treatment, while it was not in NC-H9 cells (Additional file 13: Figure S8e, upper panel). On the other hand, PP242 condition made the CCDC144NL-AS1-KD-H9 cells lose OCT4 and NANOG expression simultaneously, while NC-H9 revealed obviously reduced expression in NANOG (Additional file 13: Figure S8e, lower panel). The above results suggested that, compared to NC-H9 cells, CCDC144NL-AS1-KD-H9 cells relied more on mTORC1/2, especially mTORC2, to maintain their pluripotency.

Collectively, our results demonstrated that downregulation of *CCDC144NL-AS1* endowed human PSCs with an analogous signaling pathway dependence pattern to the naïve pluripotent stem cells, suggestive of a significant role of *CCDC144NL-AS1* in human alternative pluripotency states conversion.

## Discussion

In this study, we identify a lncRNA, *CCDC144NL-AS1*, whose downregulation drives human PSCs to a pluripotency state with characteristics resembling human naïve PSCs. *CCDC144NL-AS1*-downregulated hPSCs exhibit naïve-like dome-shaped clones, enhanced proliferation and single-cell cloning efficiency, increased developmental capacity, transcriptional profiles analogous to human naïve PSCs, genome-wide reduction of suppressive chromatin modification signals, and similar signaling pathway dependency patterns with human naïve pluripotency. Previous studies of naïve pluripotency derivation generally centered around transcription factors, small molecular inhibitors, and growth factors [14, 15, 17, 18, 65]. Only recently, two lncRNAs were reported to take part in naïve pluripotency preservation of mouse or human ESCs [77, 78]. *CCDC144NL-AS1* is the first identified lncRNA being involved in naïve-like pluripotency conversion from human primed PSCs without supplying additional transcription factors or small chemical molecules except for TeSR-E8 medium. It engenders our pondering over lncRNA roles in human naïve pluripotency regulation or early process of human embryonic development and provides new insights into the molecular switch between human primed and naïve pluripotency.

Over the last decade, lncRNAs have been functionally characterized in widespread biological processes implicating cell cycle regulation, immune response, pluripotency maintenance, lineage development, and multiple kinds of diseases, such as cancers and neurological and psychiatric disorders [29, 38, 79, 80]. Notably, thousands of known or novel lncRNAs were identified from RNA-seq data of mouse cleavage-stage embryos and human preimplantation embryos, and a large part of them exhibited developmental stage-specific expression patterns, unveiling their probable essential roles in mammalian early embryogenesis [81, 82]. From this point of view, it is not difficult to comprehend that only the modulation of a lncRNA’s expression, for instance, *CCDC144NL-AS1* knocking down here, in primed culture condition can initiate the process of human primed-to-naïve pluripotency conversion. In addition, we think the important step used to narrow down candidate lncRNAs in our screening strategy is the calculation of the membership values between pluripotency-associated genes module (PGM) and human PSCs’ enriched noncoding genes from WGCNA (Fig. 1c–d, Additional file 4: Figure S1e). This screening method enabled us to obtain the top 12 lncRNAs highly correlated with human pluripotency to conduct subsequent experiments (Fig. 1e). Hence, our study provides an efficient screening model to extract potentially useful genes from the vast RNA-seq data.

*CCDC144NL-AS1*-KD hPSCs display distinct transcriptional and epigenetic characteristics from the primed cells, but the exact underlying mechanisms are not completely understood. It is known that the usage of small molecular kinase inhibitors and growth factors in the generation of naïve pluripotency can lead to naïve-specific transcriptome and epigenome [7, 14, 17, 18]. Thus, we investigated the signaling pathways in our cells. Our data demonstrate that in CCDC144NL-AS1-KD-H9 cells, the activity of MAPK/ERK signaling pathway is inhibited, active β-catenin is accumulated, and some LIF-STAT3 target genes are upregulated, all of which are concordant with that in human naïve PSCs (Fig. 7d, e, g, h; Additional file 13: Figure S8c). Because we did not add any small molecular inhibitors to the culture conditions except for two growth factors (bFGF and TGFβ) included in TeSR E8 medium, the exact mechanisms underlying the naïve-like pluripotency conversion of human PSCs caused by *CCDC144NL-AS1* knockdown are probably different from the usual induction methods of human naïve PSCs, such as “3iL” or “5iL/A” protocols [14, 18]. In effect, a group of protein phosphatases is activated in *CCDC144NL-AS1*-KD hPSCs, such as some DUSP family members (Fig. 7e, f). We speculate that these protein phosphatases are stimulated in some way and play a crucial role in the suppression of protein kinases involved in signaling pathways. LncRNA *lincU* was reported to take part in naïve pluripotency preservation of mouse and human ESCs by binding and stabilizing DUSP9 protein and then constitutively inhibits the ERK1/2 signaling pathway [78]. Thus, *CCDC144NL-AS1* may promote the opposite processes, such as proteasome pathway or other protein degradation pathways; its knockdown then leads to the accumulation of DUSP protein and results in changes in signaling pathway activity state. On the other hand, lncRNA *lncenc1* was reported to maintain naïve states of mouse ESCs by promoting the glycolysis pathway [77]. GO classification and enrichment analyses also revealed that our CCDC144NL-AS1-KD-hPSCs were highly correlated with energy generation terms (Fig. 5c, upper panel). So, *CCDC144NL-AS1* may also play roles in this process. Additionally, we found totally four lncRNAs in the first lncRNA screening part. Our examination results of the expression changes of the other three lncRNAs after *CCDC144NL-AS1* knockdown in H9 showed that *LOC100506930*-1 (NR_038278) and *LOC100506930*-2 (NR_038279) were significantly increased (Additional file 13: Figure S8f). Hence, *LOC100506930*-1 (NR_038278) and *LOC100506930*-2 (NR_038279) may be involved in the pluripotency state conversion of hPSCs triggered by *CCDC144NL-AS1* downregulation. Generally speaking, our *CCDC144NL-AS1*-KD hPSCs provide a platform to further investigate what other kinds of intermediate regulators are involved in human early pluripotency regulation.

Of note, some expression of *CCDC144NL-AS1* is required in naïve-like type hPSCs. The expression of *CCDC144NL-AS1* in H9 cells was hundreds-fold higher than that in HEF (Fig. 1g) and was about 2.5-fold higher than that in CCDC144NL-AS1-KD-H9 (Fig. 2f). The *CCDC144NL-AS1* mRNA levels in CCDC144NL-AS1-KD-hESCs was obviously higher than that in HEF. Actually, the expression data of *CCDC144NL-AS1* in naïve H9 and its control H9 cells from Takashima et al. [15], and in 55 samples we used in the lncRNA screening process which contained 21 hiPSC samples, 15 hESC samples, and 19 human somatic tissue samples, revealed that *CCDC144NL-AS1* was expressed significantly lower in naïve H9 than that in human PSCs, but obviously higher than that in human somatic tissues (Additional file 13: Figure S8 g).

Previous’ studies and our research all suggest that the acquisition of naïve pluripotency involves a quite intricate regulatory network, but a substantial part of it is still undiscovered. Our study unveils a lncRNA’s unexpected role in the complex network of naïve-like pluripotency transition from human PSCs, supplies a new perspective to further understand the lncRNA’s effects in the early process of human embryonic development, and provides a platform to decipher unknown aspects of human naïve pluripotency. We think that *CCDC144NL-AS1* can be potentially valuable for future research on deriving higher quality naïve state hPSCs and promoting their therapeutic applications.

## Conclusions

*CCDC144NL-AS1* knockdown induces naïve-like state conversion of hPSCs without suppling transcription factors or small molecular inhibitors. CCDC144NL-AS1-KD hPSCs exhibit dome-shaped clones, increased developmental capacity, transcriptional profiles analogous to human naïve PSCs, genome-wide reduction of suppressive chromatin modification signals, and similar signaling pathway dependency patterns with human naïve pluripotency. Our study supplies a new perspective to further understand the lncRNA’s effects in the early process of human embryonic development and provides a platform to decipher unknown aspects of human naïve pluripotency.

## Additional files

Additional file 1: **Table S1.** Summary of primer sequences for quantitative real-time PCR in this study. (XLSX 12 kb)

Additional file 2: **Table S2.** Antibodies used in immunofluorescence staining and western blot analysis. (XLSX 11 kb)

Additional file 3: **Table S3.** RNA-seq datasets utilized in this paper for screening lncRNAs that were specifically highly expressed in human PSCs. (XLSX 10 kb)

Additional file 4: **Figure S1.** Functional categories of genes from three human PSC-specific modules enriched by WGCNA. a WGCNA dendogram indicating expression of different gene modules in all 55 samples. b-d Gene Ontology (GO) and Kyoto Encyclopedia of Genes and Genomes (KEGG) analyses of genes in MEred (b), MElightcyan (c), and MEpurple (d) modules. e List of pluripotency-associated genes in PGM module. (PDF 3714 kb)

Additional file 5: **Video S1.** Confocal laser scanning of CCDC144NL-AS1-KD-H9 cluster accompanied by 90° horizontal rotation. (MP4 1823 kb)

Additional file 6: **Video S2.** Confocal laser scanning of NC-H9 cluster accompanied by 90° horizontal rotation. (MP4 1745 kb)

Additional file 7: **Figure S2.** Pluripotency validation of *CCDC144NL-AS1* downregulated H14 and HDF-iPS cells. a-d Quantitative RT-PCR analyses of three core pluripotency and naïve pluripotency genes (a, c) and lineage commitment factor genes (b, d) in CCDC144NL-AS1-KD-H14, NC-H14, CCDC144NL-AS1-KD-HDF-iPS, and NC-HDF-iPS cells. Error bars indicate SEM (*n* = 3). *p < .05; **p < .01; ***p < .001. e-f Immunostaining images of pluripotency-associated markers OCT4, NANOG, SOX2, SSEA3/4, and TRA-1-60 (e) and naïve pluripotency-specific marker TFCP2L1 (f) for CCDC144NL-AS1-KD-H14, NC-H14, CCDC144NL-AS1-KD-HDF-iPS, and NC-HDF-iPS cells. Scale bars, 100 μm. g Western blot detection of naïve pluripotency-related transcription factors, such as NANOG and KLF4, and shared pluripotency transcription factors, such as OCT4 and SOX2, in CCDC144NL-AS1-KD-H14, NC-H14, CCDC144NL-AS1-KD-HDF-iPS, and NC-HDF-iPS cells. β-Tubulin was used as an endogenous control. (PNG 860 kb)

Additional file 8: **Figure S3.** Cell growth pattern analysis of *CCDC144NL-AS1*-downregulated H9, H14, and HDF-iPS cells. a Single-cell cloning efficiencies of CCDC144NL-AS1-KD-H14, NC-H14, CCDC144NL-AS1-KD-HDF-iPS, and NC-HDF-iPS cells. Error bars indicate SEM (*n* = 3); **p < .01. b-c After plating cells for 4 days, the cell cycle of CCDC144NL-AS1-KD-H9 and NC-H9 was analyzed by flow cytometry. The representative flow cytometry analysis results (b) and the percentages of cells in G0/G1 and S+G2/M phases (c) are shown. d-g After plating cells for 4 days, the cell cycle of CCDC144NL-AS1-KD-H14, NC-H14, CCDC144NL-AS1-KD-HDF-iPS, and NC-HDF-iPS cells was analyzed by flow cytometry. The representative flow cytometry analysis results (d, f) and the percentages of cells in G0/G1 and S+G2/M phases (e, g) are shown. (PDF 660 kb)

Additional file 9: **Figure S4.** Developmental potential of *CCDC144NL-AS1*-downregulated H14 and HDF-iPS cells in vitro. a Morphologies of CCDC144NL-AS1-KD-H9- and NC-H9-derived neuronal, endodermal, and mesodermal cells. b, f, l Quantitative RT-PCR analyses of expression levels of lineage-specific genes in CCDC144NL-AS1-KD-HDF-iPS and NC-HDF-iPS-derived neuronal (b), endodermal (f), and mesodermal (l) cells. Error bars indicate SEM (*n* = 3). *p < .05; **p < .01; ***p < .001. c, g, m Western blot analyses of lineage-specific genes’ expression in CCDC144NL-AS1-KD-HDF-iPS- and NC-HDF-iPS-derived neuronal (c), endodermal (g), and mesodermal (m) cells. β-Tubulin was used as an endogenous control. d, h, j Quantitative RT-PCR analyses of expression levels of lineage-specific genes in CCDC144NL-AS1-KD-H14- and NC-H14-derived neuronal (d), endodermal (h), and mesodermal (j) cells. Error bars indicate SEM (*n* = 3). *p < .05; **p < .01; ***p < .001. e, i, k Western blot analyses of lineage-specific genes’ expression in CCDC144NL-AS1-KD-H14- and NC-H14-derived neuronal (e), endodermal (i), and mesodermal (k) cells. β-Tubulin was used as an endogenous control. (PNG 1548 kb)

Additional file 10: **Figure S5.** Embryoid body formation analysis of *CCDC144NL-AS1*-downregulated H14 and HDF-iPS. a Quantitative RT-PCR analyses of cell adhesion-associated genes in embryoid bodies generated from CCDC144NL-AS1-KD-H9 and NC-H9 cells. Error bars indicate SEM (*n* = 3). *p < .05; **p < .01; ***p < .001. b-e Quantitative RT-PCR analyses of lineage-related genes in embryoid bodies generated from CCDC144NL-AS1-KD-H14 (b), NC-H14 (c), CCDC144NL-AS1-KD-HDF-iPS (d), and NC-HDF-iPS (e) cells. Error bars indicate SEM (*n* = 3). *p < .05; **p < .01; ***p < .001. f-g Embryoid body numbers generated from per 10^6^ CCDC144NL-AS1-KD-H14 and NC-H14 (f) and CCDC144NL-AS1-KD-HDF-iPS and NC-HDF-iPS (g) cells. Error bars indicate SEM (*n* = 3). *p < .05; **p < .01. (PDF 448 kb)

Additional file 11: **Figure S6.** a-b Representative immunostaining images of three embryonic germ layer markers for teratomas derived from CCDC144NL-AS1-KD-H9 (a) and NC-H9 (b) cells. β III TUBULIN stands for ectoderm, E-CADHERIN stands for endoderm, and VIMENTIN represents mesoderm. Scale bars, 100 μm. c Hematoxylin and eosin staining of teratomas containing tissues of all three embryonic germ layers derived from CCDC144NL-AS1-KD-H14, NC-H14, CCDC144NL-AS1-KD-HDF-iPS, and NC-HDF-iPS cells. f, I, l, o Gut-like epithelium (endoderm). g, j, m, p Cartilage (mesoderm). h, k, n, q Neural tissue (ectoderm). Scale bars, 50 μm. d-e Teratoma formation rate of CCDC144NL-AS1-KD-H14 and NC-H14 cells (d) and CCDC144NL-AS1-KD-HDF-iPS and NC-HDF-iPS cells (e). Error bars indicate SEM (*n* = 6). *p < .05. (PNG 3193 kb)

Additional file 12: **Figure S7.** Functional categories of genes from CCDC144NL-AS1-KD human PSC-specific MEblue module and upregulated enrichment of respiratory chain in CCDC144NL-AS1-KD-H9 by GSEA. a GO analyses of MEblue module genes. b The enriched upregulated respiratory chain by gene set enrichment analysis (GSEA) in CCDC144NL-AS1-KD-H9 cells relative to NC-H9 cells. Vertical black bars represent the position of genes involved in the respiratory chain. (PDF 439 kb)

Additional file 13: **Figure S8.** Self-renewal of CCDC144NL-AS1-KD hESCs is TGF-β and mTORC1/2 dependent and exhibit relative activated LIF-STAT3 signaling pathway state. a The relative NANOG protein intensity of western blot analyses in CCDC144NL-AS1-rescued CCDC144NL-AS1-KD-H9 and their control cells. β-Tubulin was used as an endogenous control. Error bars indicate SEM (*n* = 3). **p < .01. b Images of differentiated cell morphologies of CCDC144NL-AS1-KD-H9 and NC-H9 after adding SB 431542 into the culture medium for 7 days. Scale bars, 100 μm. c Heatmap of genes implicated in LIF-STAT3 signaling pathway in CCDC144NL-AS1-KD hPSCs and control cells. The expression levels of genes are determined by RNA-seq data. d Images of NC-H9 and CCDC144NL-AS1-KD-H9 cell morphologies after 48 h treatment of rapamycin (6 μM) and PP242 (2.5 μM). Scale bars, 100 μm. e Immunostaining images of pluripotency-associated markers OCT4 and NANOG for NC-H9 and CCDC144NL-AS1-KD-H9 cells after 48 h treatment of rapamycin (6 μM) and PP242 (2.5 μM). Scale bars, 100 μm. f Quantitative RT-PCR analyses of *LOC100506930*-1 (NR_038278), *LOC100506930*-2 (NR_038279), and DDX11-AS1 in CCDC144NL-AS1-KD-H9 and NC-H9 cells. **p < .01. g The expression data of *CCDC144NL-AS1* in naïve H9 and its control H9 cells from Takashima et al. [15], and in 55 samples, we used in the lncRNA screening process which contained 21 hiPSC samples, 15 hESC samples, and 19 human somatic tissue samples. Displayed are FPKM values. (PNG 1613 kb)

## Data Availability

All data generated or analyzed during this study are included in this published article. Data of RNA-seq and ChIP-seq inthis study have been submitted to the NCBI Gene Expression Omnibus (GEO;  http://www.ncbi.nlm.nih.gov/geo/) under accession number GSE111929.

## Abbreviations

bFGF: Basic fibroblast growth factor
ChIP-Seq: Chromatin immunoprecipitation followed by deep sequencing
DUSP: Dual-specificity phosphatase
EBs: Embryoid bodies
EpiSCs: Post-implantation epiblast-derived stem cells
FGF2: Fibroblast growth factor 2
FGFR: Fibroblast growth factor receptor
FPKM: Fragments per kilobase of transcript per million fragments mapped
GO: Gene Ontology
GSEA: Gene set enrichment analysis
GSK3: Glycogen synthase kinase-3
HEF: Human embryonic fibroblast
hESCs: Human embryonic stem cells
hiPSCs: Human induced pluripotent stem cells
HNES: Human naïve embryonic stem
hPSCs: Human pluripotent stem cells
KD: Knockdown
KEGG: Kyoto Encyclopedia of Genes and Genomes
KLF4: Kruppel-like factor 4
LIF: Leukemia inhibitory factor
lncRNAs: Long noncoding RNAs
MAPK or ERK: Mitogen-activated protein kinase
MAPKK or MEK: Mitogen-activated protein kinase kinase
NOD/SCID: Non-obese diabetic severe combined immunodeficiency
NPCs: Neural precursor cells
OCT4: Octamer-binding protein 4
PGM: Pluripotency-associated gene module
PSCs: Pluripotent stem cells
qPCR: Quantitative real-time PCR
ROCK: Rho-associated protein kinase
RPKM: Reads per kilobase per million mapped reads
shRNAs: Short hairpin RNAs
TCF3: Transcription factor 3
TGF-β: Transforming growth factor beta
TOM: Topological overlap measure
TSS: Transcription start site
WGCNA: Weighted gene co-expression network analysis
ZIC: Zinc finger of the cerebellum
