# Supplementary material for: Long noncoding RNA CCDC144NL-AS1 knockdown induces naïve-like state conversion of human pluripotent stem cells
Source: Stem Cell Res Ther. 2019 Jul 29;10:220. doi: 10.1186/s13287-019-1323-9 (PMC6664583; doi:10.1186/s13287-019-1323-9)
Supplement: Supplementary file 4 — Figure S1. Functional categories of genes from three human PSC-specific modules enriched by WGCNA. a WGCNA dendogram indicating expression of different gene modules in all 55 samples. b-d Gene Ontology (GO) and Kyoto Encyclopedia of Genes and Genomes (KEGG) analyses of genes in MEred (b), MElightcyan (c), and MEpurple (d) modules. e List of pluripotency-associated genes in PGM module. (PDF 3714 kb) [file 13287_2019_1323_MOESM4_ESM.pdf]

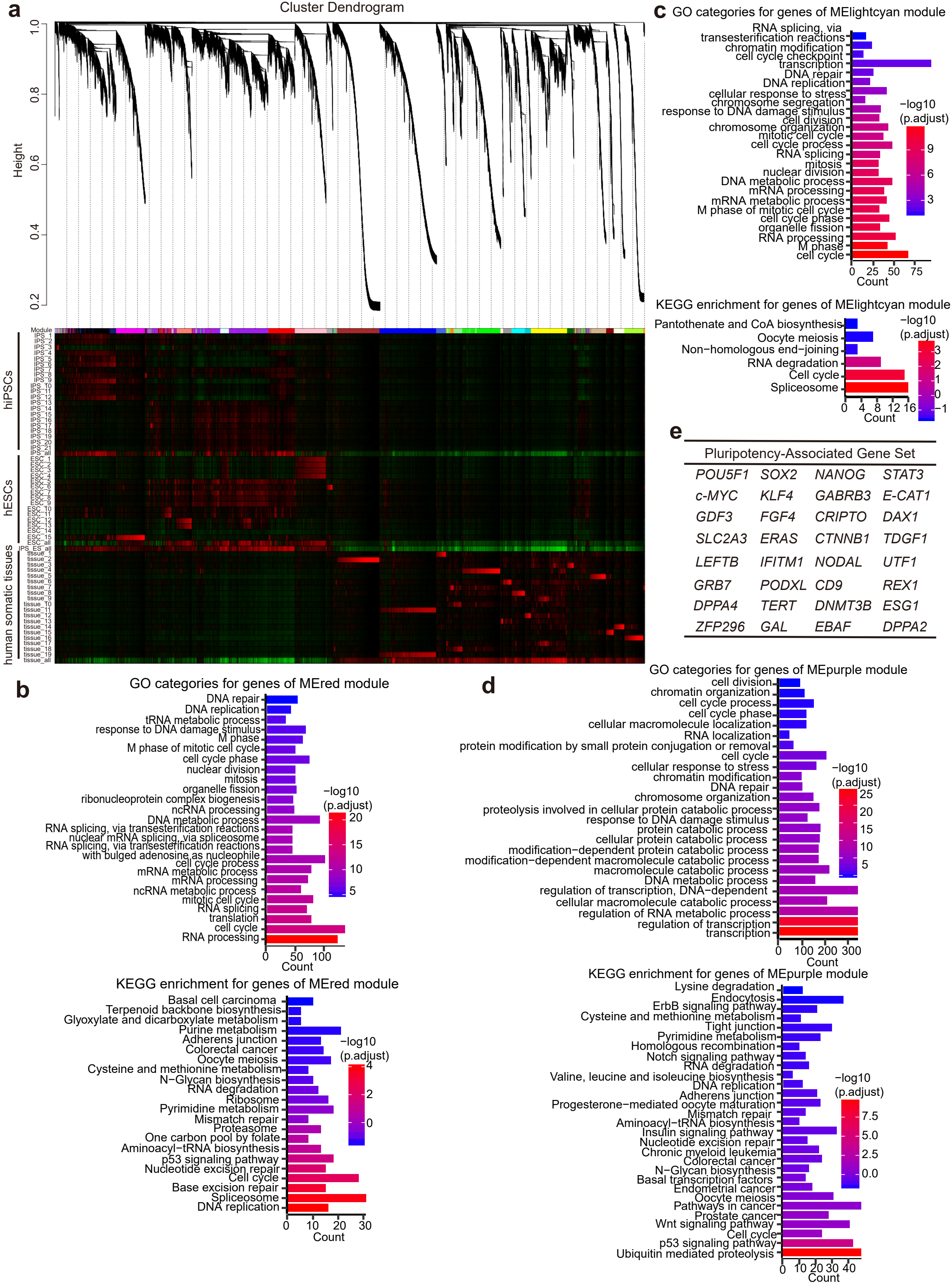

e

Pluripotency-Associated Gene Set			
POU5F1	*SOX2*	*NANOG*	*STAT3*
c-MYC	*KLF4*	*GABRB3*	*E-CAT1*
GDF3	*FGF4*	*CRIPTO*	*DAX1*
SLC2A3	*ERAS*	*CTNNB1*	*TDGF1*
LEFTB	*IFITM1*	*NODAL*	*UTF1*
GRB7	*PODXL*	*CD9*	*REX1*
DPPA4	*TERT*	*DNMT3B*	*ESG1*
ZFP296	*GAL*	*EBAF*	*DPPA2*
