# Supplementary figures and images for: Long noncoding RNA CCDC144NL-AS1 knockdown induces naïve-like state conversion of human pluripotent stem cells
Source: Stem Cell Res Ther. 2019 Jul 29;10:220. doi: 10.1186/s13287-019-1323-9 (PMC6664583; doi:10.1186/s13287-019-1323-9)

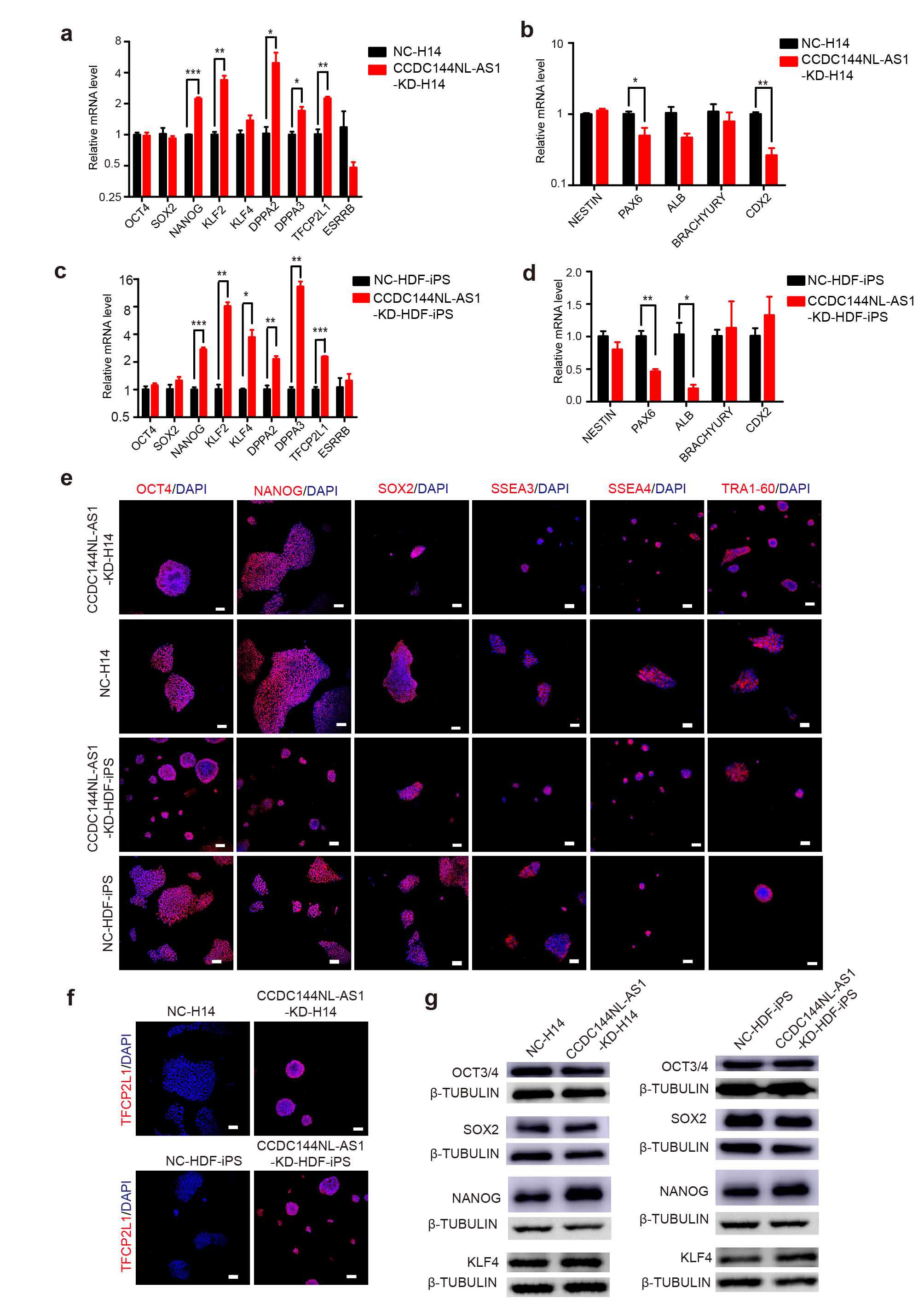

Supplement: Supplementary file 7 — Figure S2. Pluripotency validation of CCDC144NL-AS1 downregulated H14 and HDF-iPS cells. a-d Quantitative RT-PCR analyses of three core pluripotency and naïve pluripotency genes (a, c) and lineage commitment factor genes (b, d) in CCDC144NL-AS1-KD-H14, NC-H14, CCDC144NL-AS1-KD-HDF-iPS, and NC-HDF-iPS cells. Error bars indicate SEM (n = 3). *p < .05; **p < .01; ***p < .001. e-f Immunostaining images of pluripotency-associated markers OCT4, NANOG, SOX2, SSEA3/4, and TRA-1-60 (e) and naïve pluripotency-specific marker TFCP2L1 (f) for CCDC144NL-AS1-KD-H14, NC-H14, CCDC144NL-AS1-KD-HDF-iPS, and NC-HDF-iPS cells. Scale bars, 100 μm. g Western blot detection of naïve pluripotency-related transcription factors, such as NANOG and KLF4, and shared pluripotency transcription factors, such as OCT4 and SOX2, in CCDC144NL-AS1-KD-H14, NC-H14, CCDC144NL-AS1-KD-HDF-iPS, and NC-HDF-iPS cells. β-Tubulin was used as an endogenous control. (PNG 860 kb) [file 13287_2019_1323_MOESM7_ESM.png]

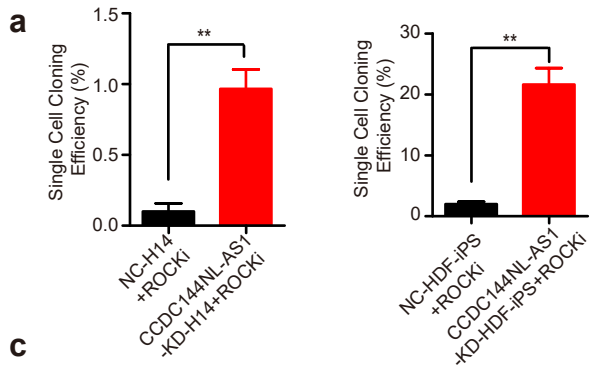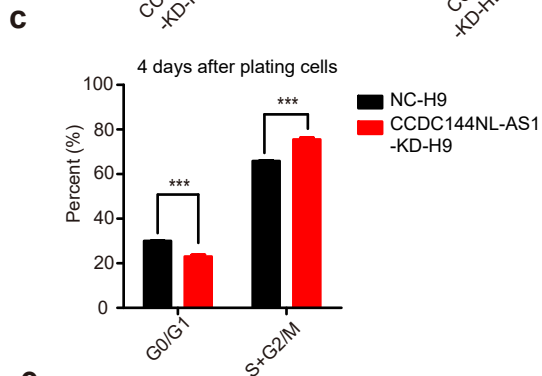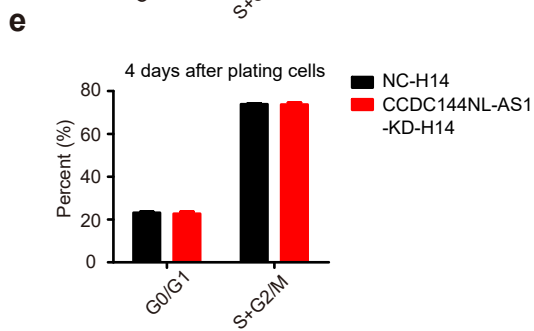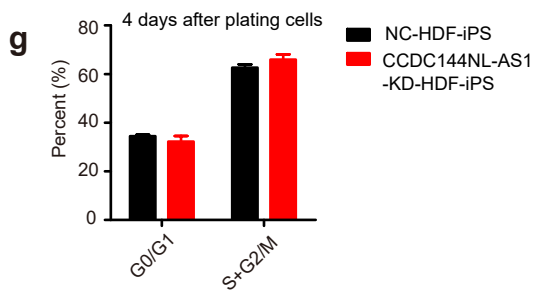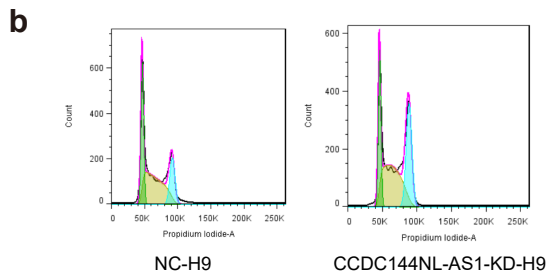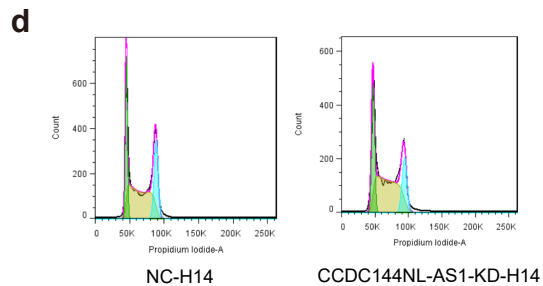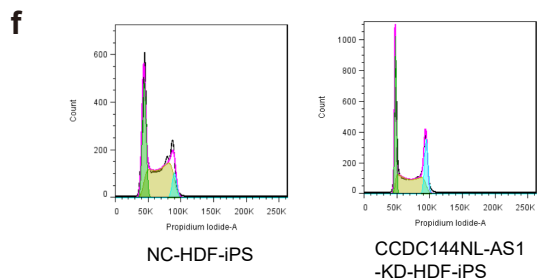

Supplement: Supplementary file 8 — Figure S3. Cell growth pattern analysis of CCDC144NL-AS1-downregulated H9, H14, and HDF-iPS cells. a Single-cell cloning efficiencies of CCDC144NL-AS1-KD-H14, NC-H14, CCDC144NL-AS1-KD-HDF-iPS, and NC-HDF-iPS cells. Error bars indicate SEM (n = 3); **p < .01. b-c After plating cells for 4 days, the cell cycle of CCDC144NL-AS1-KD-H9 and NC-H9 was analyzed by flow cytometry. The representative flow cytometry analysis results (b) and the percentages of cells in G0/G1 and S+G2/M phases (c) are shown. d-g After plating cells for 4 days, the cell cycle of CCDC144NL-AS1-KD-H14, NC-H14, CCDC144NL-AS1-KD-HDF-iPS, and NC-HDF-iPS cells was analyzed by flow cytometry. The representative flow cytometry analysis results (d, f) and the percentages of cells in G0/G1 and S+G2/M phases (e, g) are shown. (PDF 660 kb) [file 13287_2019_1323_MOESM8_ESM.pdf]

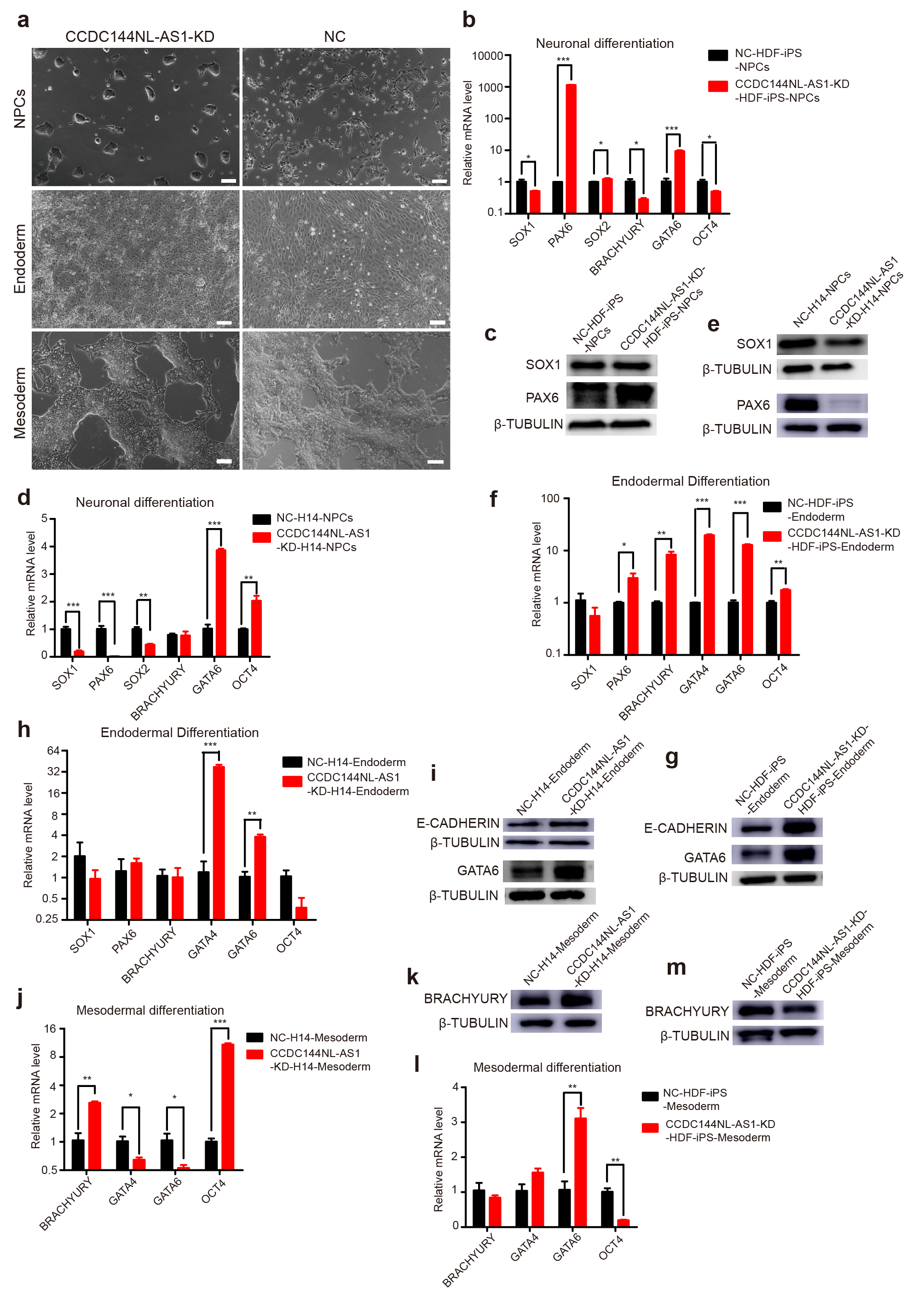

Supplement: Supplementary file 9 — Figure S4. Developmental potential of CCDC144NL-AS1-downregulated H14 and HDF-iPS cells in vitro. a Morphologies of CCDC144NL-AS1-KD-H9- and NC-H9-derived neuronal, endodermal, and mesodermal cells. b, f, l Quantitative RT-PCR analyses of expression levels of lineage-specific genes in CCDC144NL-AS1-KD-HDF-iPS and NC-HDF-iPS-derived neuronal (b), endodermal (f), and mesodermal (l) cells. Error bars indicate SEM (n = 3). *p < .05; **p < .01; ***p < .001. c, g, m Western blot analyses of lineage-specific genes’ expression in CCDC144NL-AS1-KD-HDF-iPS- and NC-HDF-iPS-derived neuronal (c), endodermal (g), and mesodermal (m) cells. β-Tubulin was used as an endogenous control. d, h, j Quantitative RT-PCR analyses of expression levels of lineage-specific genes in CCDC144NL-AS1-KD-H14- and NC-H14-derived neuronal (d), endodermal (h), and mesodermal (j) cells. Error bars indicate SEM (n = 3). *p < .05; **p < .01; ***p < .001. e, i, k Western blot analyses of lineage-specific genes’ expression in CCDC144NL-AS1-KD-H14- and NC-H14-derived neuronal (e), endodermal (i), and mesodermal (k) cells. β-Tubulin was used as an endogenous control. (PNG 1548 kb) [file 13287_2019_1323_MOESM9_ESM.png]

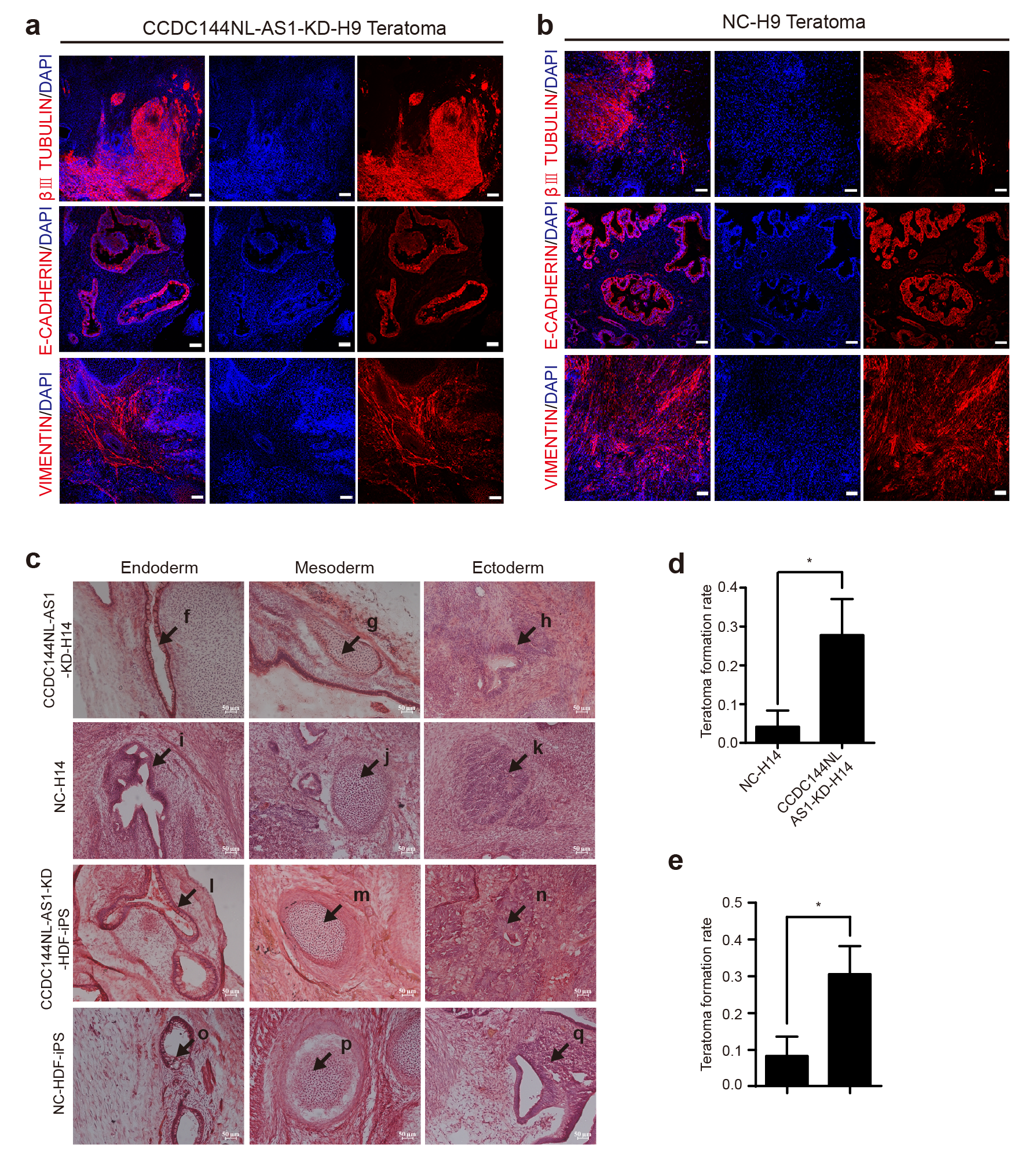

Supplement: Supplementary file 11 — Figure S6. a-b Representative immunostaining images of three embryonic germ layer markers for teratomas derived from CCDC144NL-AS1-KD-H9 (a) and NC-H9 (b) cells. β III TUBULIN stands for ectoderm, E-CADHERIN stands for endoderm, and VIMENTIN represents mesoderm. Scale bars, 100 μm. c Hematoxylin and eosin staining of teratomas containing tissues of all three embryonic germ layers derived from CCDC144NL-AS1-KD-H14, NC-H14, CCDC144NL-AS1-KD-HDF-iPS, and NC-HDF-iPS cells. f, I, l, o Gut-like epithelium (endoderm). g, j, m, p Cartilage (mesoderm). h, k, n, q Neural tissue (ectoderm). Scale bars, 50 μm. d-e Teratoma formation rate of CCDC144NL-AS1-KD-H14 and NC-H14 cells (d) and CCDC144NL-AS1-KD-HDF-iPS and NC-HDF-iPS cells (e). Error bars indicate SEM (n = 6). *p < .05. (PNG 3193 kb) [file 13287_2019_1323_MOESM11_ESM.png]

**a**

### GO categories for genes of MEblue module

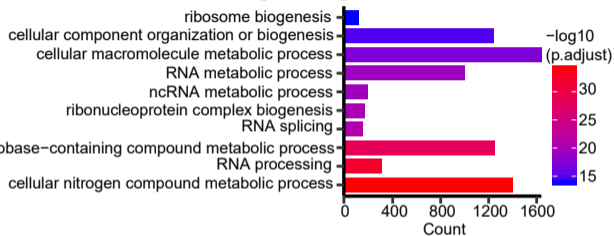**b**

### RESPIRATORY CHAIN

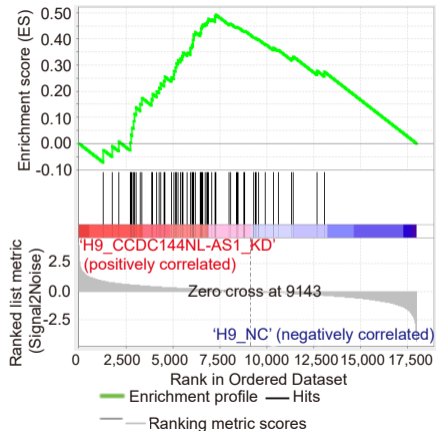

Supplement: Supplementary file 12 — Figure S7. Functional categories of genes from CCDC144NL-AS1-KD human PSC-specific MEblue module and upregulated enrichment of respiratory chain in CCDC144NL-AS1-KD-H9 by GSEA. a GO analyses of MEblue module genes. b The enriched upregulated respiratory chain by gene set enrichment analysis (GSEA) in CCDC144NL-AS1-KD-H9 cells relative to NC-H9 cells. Vertical black bars represent the position of genes involved in the respiratory chain. (PDF 439 kb) [file 13287_2019_1323_MOESM12_ESM.pdf]

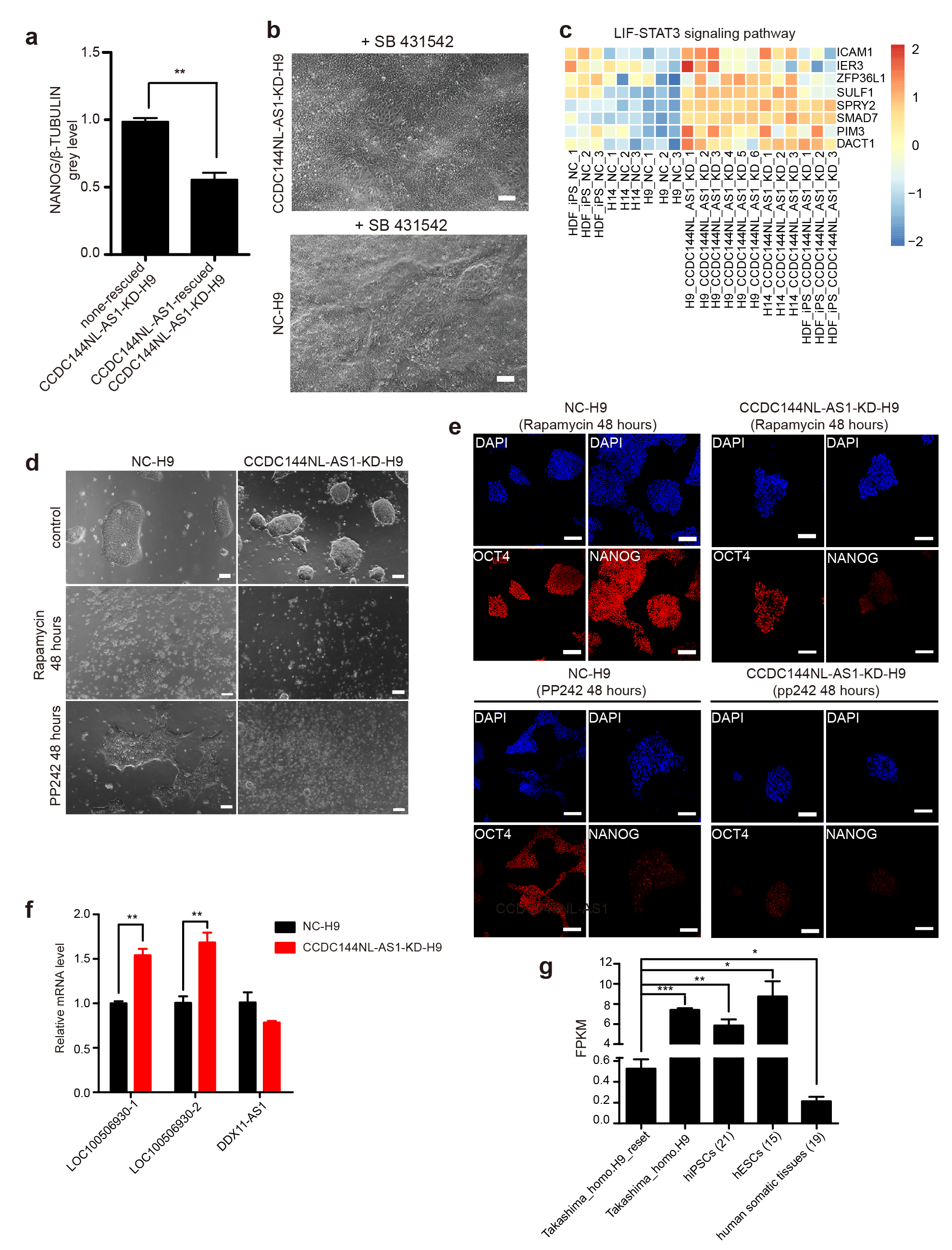

Supplement: Supplementary file 13 — Figure S8. Self-renewal of CCDC144NL-AS1-KD hESCs is TGF-β and mTORC1/2 dependent and exhibit relative activated LIF-STAT3 signaling pathway state. a The relative NANOG protein intensity of western blot analyses in CCDC144NL-AS1-rescued CCDC144NL-AS1-KD-H9 and their control cells. β-Tubulin was used as an endogenous control. Error bars indicate SEM (n = 3). **p < .01. b Images of differentiated cell morphologies of CCDC144NL-AS1-KD-H9 and NC-H9 after adding SB 431542 into the culture medium for 7 days. Scale bars, 100 μm. c Heatmap of genes implicated in LIF-STAT3 signaling pathway in CCDC144NL-AS1-KD hPSCs and control cells. The expression levels of genes are determined by RNA-seq data. d Images of NC-H9 and CCDC144NL-AS1-KD-H9 cell morphologies after 48 h treatment of rapamycin (6 μM) and PP242 (2.5 μM). Scale bars, 100 μm. e Immunostaining images of pluripotency-associated markers OCT4 and NANOG for NC-H9 and CCDC144NL-AS1-KD-H9 cells after 48 h treatment of rapamycin (6 μM) and PP242 (2.5 μM). Scale bars, 100 μm. f Quantitative RT-PCR analyses of LOC100506930-1 (NR_038278), LOC100506930-2 (NR_038279), and DDX11-AS1 in CCDC144NL-AS1-KD-H9 and NC-H9 cells. **p < .01. g The expression data of CCDC144NL-AS1 in naïve H9 and its control H9 cells from Takashima et al. [15], and in 55 samples, we used in the lncRNA screening process which contained 21 hiPSC samples, 15 hESC samples, and 19 human somatic tissue samples. Displayed are FPKM values. (PNG 1613 kb) [file 13287_2019_1323_MOESM13_ESM.png]
