# Supplementary material for: Long noncoding RNA CCDC144NL-AS1 knockdown induces naïve-like state conversion of human pluripotent stem cells
Source: Stem Cell Res Ther. 2019 Jul 29;10:220. doi: 10.1186/s13287-019-1323-9 (PMC6664583; doi:10.1186/s13287-019-1323-9)
Supplement: Supplementary file 10 — Figure S5. Embryoid body formation analysis of CCDC144NL-AS1-downregulated H14 and HDF-iPS. a Quantitative RT-PCR analyses of cell adhesion-associated genes in embryoid bodies generated from CCDC144NL-AS1-KD-H9 and NC-H9 cells. Error bars indicate SEM (n = 3). *p < .05; **p < .01; ***p < .001. b-e Quantitative RT-PCR analyses of lineage-related genes in embryoid bodies generated from CCDC144NL-AS1-KD-H14 (b), NC-H14 (c), CCDC144NL-AS1-KD-HDF-iPS (d), and NC-HDF-iPS (e) cells. Error bars indicate SEM (n = 3). *p < .05; **p < .01; ***p < .001. f-g Embryoid body numbers generated from per 106 CCDC144NL-AS1-KD-H14 and NC-H14 (f) and CCDC144NL-AS1-KD-HDF-iPS and NC-HDF-iPS (g) cells. Error bars indicate SEM (n = 3). *p < .05; **p < .01. (PDF 448 kb) [file 13287_2019_1323_MOESM10_ESM.pdf]

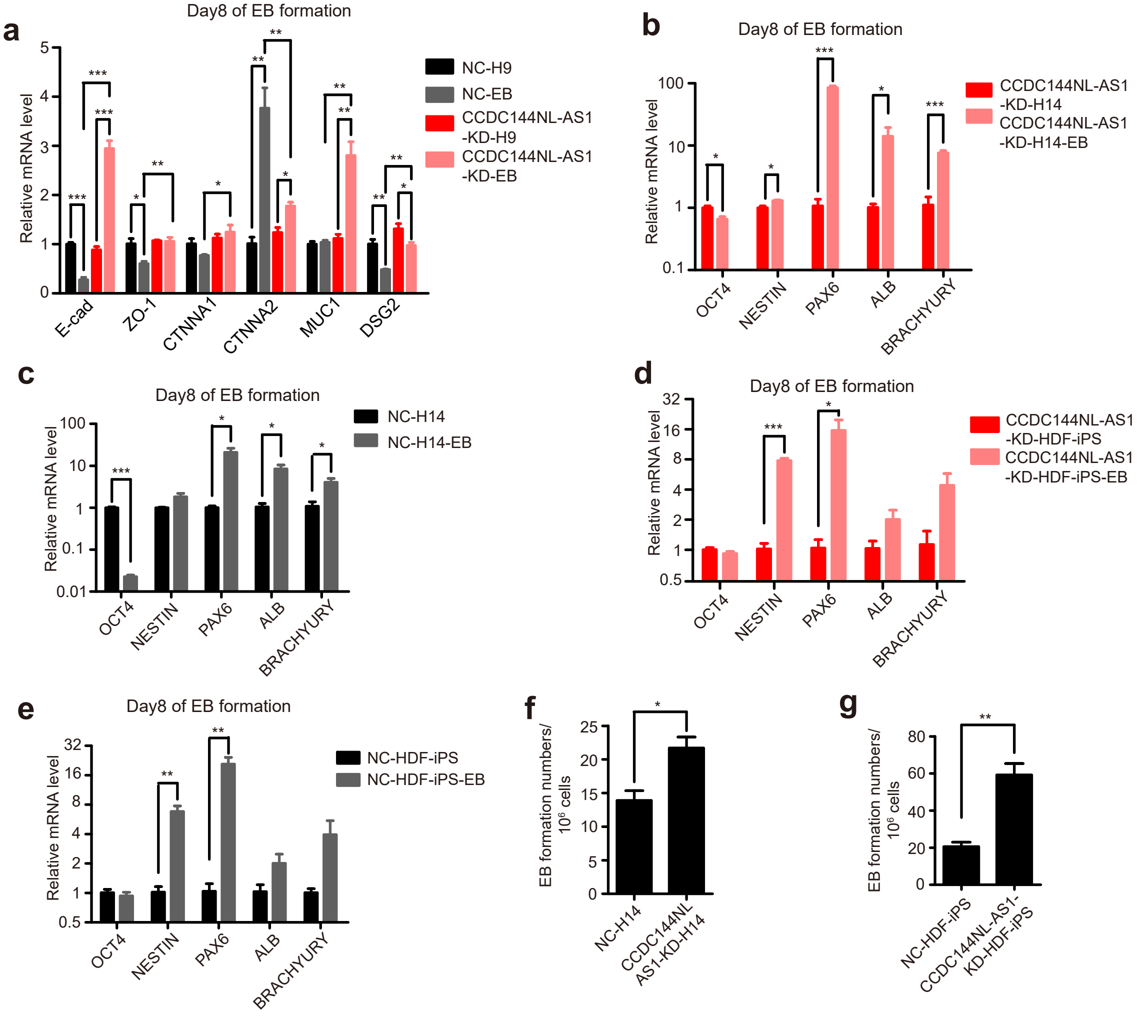

a
Day8 of EB formation
Relative mRNA level
NC-H9
NC-EB
CCDC144NL-AS1 -KD-H9
CCDC144NL-AS1 -KD-EB
E-cad
ZO-1
CTNNA1
CTNNA2
MUC1
DSG2
b
Day8 of EB formation
Relative mRNA level
CCDC144NL-AS1 -KD-H14
CCDC144NL-AS1 -KD-H14-EB
OCT4
NESTIN
PAX6
ALB
BRACHYURY
c
Day8 of EB formation
Relative mRNA level
NC-H14
NC-H14-EB
d
Day8 of EB formation
Relative mRNA level
CCDC144NL-AS1 -KD-HDF-iPS
CCDC144NL-AS1 -KD-HDF-iPS-EB
e
Day8 of EB formation
Relative mRNA level
NC-HDF-iPS
NC-HDF-iPS-EB
f
EB formation numbers/ 10^6 cells
NC-H14
CCDC144NL AS1-KD-H14
g
EB formation numbers/ 10^6 cells
NC-HDF-iPS
CCDC144NL-AS1- KD-HDF-iPS
